# Comparison of radiant intensity in aqueous media using experimental and numerical simulation techniques

**DOI:** 10.12688/openreseurope.16812.1

**Published:** 2024-01-11

**Authors:** Adithya Pai Uppinakudru, Cintia Casado, Ken Reynolds, Simon Stanley, Cristina Pablos, Javier Marugán

**Affiliations:** 1 Universidad Rey Juan Carlos, Móstoles, Community of Madrid, Spain; 2 Prophotonix, Cork, Ireland

**Keywords:** actinometry, discrete ordinate, optical design, radiometry, ray tracing, ultraviolet light

## Abstract

**Background:**

Measurement of light intensity reaching a point of interest in complex systems is a challenge faced by academia and industry. This study analyzes an optical ray tracing method to predict the radiant intensity reaching a point of interest in a germicidal system.

**Methods:**

Implementation was performed by analyzing how the method compares with the discrete ordinate method, radiometry, and actinometry. This study further quantified the effect of the photoreactor quartz tube on the measured intensity for multiple wavelengths.

**Results:**

Light intensity losses were estimated to be 10 ± 0.5% for the FX-1 265 source. In contrast, the simulation in a water medium showed an increase of up to 64% in the light intensity delivered to the central part of the tube owing to internal reflections and scattering. Model predictions from ray tracing were successfully compared with the discrete ordinate method (DOM) and experimental data (within ± 6%), ensuring the accurate design of complex systems for water disinfection.

**Conclusions:**

The data from simulations address the challenges faced in complex radiation modeling and demonstrate that the method can be utilized as a useful tool for optimization and prediction.

## Plain language summary

The following study explores a new way to measure the strength of light in complex systems, particularly in water treatment type setups. We used a method called optical ray tracing to predict how much light reaches a specific point in the system and have compared it with other techniques like radiometry and discrete ordinate method to make sure it is valid and accurate. An interesting finding was that the type of tube material, in this case, a quartz tube, affects the intensity of the light. For a specific light source with 265 nm light emitting diodes, we estimate a 10% reduction in light intensity due to the quartz tube (transporter of water through the system) in air. But when the simulation was done in a water medium, we found a 64% increase in light intensity in the center of the tube because of how light bounces around and scatters in water. The optical ray tracing method's predictions closely matched other techniques (within about 6% of error), making it a useful tool for designing systems for water disinfection. The study also builds a complex system and predicts the amount of light reaching certain points of interest. This research helps tackle challenges in modeling complex radiation systems and offers insights into how to optimize and predict the behavior of light in such systems.

**Table:** 

**Nomenclature**			
***Abbreviations***		***Symbols***	
**DOM**	Discrete Ordinate Method	**A (λ),** ***abs***	Decadic absorbance (dimensionless)
**IUPAC**	International Union of Pure and Applied Chemistry	**q ^0^_p_**	incident photon flux
**LED**	Light Emitting Diodes	***k***	Ray direction cosine vector
**PCB**	Printed Circuit Board	***l, m, n***	Direction cosines of unit vector $\vec{k}$
**RTE**	Radiative Transfer Equation	**N**	Unit normal vector of the surface at the point of contact
**UV**	Ultraviolet light in the wavelength range of 100- 400 nm	**T**	Temperature (K)
		***x, y, z***	Coordinates of ray (m)
***Greek Letters***		***Vector Symbols***	
**λ**	Wavelength (nm)	***I*_λ,_**_ $\vec{\Omega }$ _	Intensity of photons with wavelength λ and direction $\vec{\Omega }$
**ρ **	Density (kg m ^-3^)	$\hat{k}$	Direction of the ray
**σ**	Stefan Boltzmann constant (W m ^-2^ K ^-4^)	$\vec{\Omega }$	Unit vector in the direction of radiation propagation
**σ _k_**	Volumetric scattering coefficient (m ^-1^)	$\vec{r}$	Directional position of the ray in a cartesian coordinate system
**Φ**	Wavelength averaged primary quantum yield (mol Einstein ^-1^)		
**Φ(λ)**	Number of molecules changed, formed or destroyed divided by number of absorbed photons		
***Ω***	Solid angle of radiation propagation about the direction $\vec{\Omega }$		

## Introduction

The recent increase in demand for and interest in ultraviolet (UV) light devices for disinfection has led to increased research to optimize the process and achieve better disinfection rates ^1^. One key aspect of evaluating and optimizing the efficiency of a UV reactor is understanding the path of light as it travels through the system. Many methods have been applied theoretically and experimentally to understand the path of ultraviolet light as it travels through a specific medium, since the idea of its germicidal effectiveness was observed by Downes *et al.* in 1877 ^2^. Experimental techniques, such as radiometry and actinometry, are physical techniques that require the use of a physical validation setup for light measurements ^3^. Radiometry is widely used in the lighting industry for measurement of light sources ^3^. This technique employs a radiometer (consisting of a sensor or detector and a signal processing unit), which measures the amount of light reaching its receiving surface when subjected to light. While it is very useful in air media, it faces challenges in water-based environments. Very few detectors and radiometers exist in the market that are waterproof or can measure light in water ^4– 6^. These detectors are expensive and/or require special attachments, which could cause other issues such as leaks, inaccurate measurements etc., within the system ^7^. Chemical actinometry is another experimental tool used in laboratories. This technique involves the use of chemicals that absorb photons as light passes through the system, leading to a measurable chemical reaction from which the number of photons absorbed is estimated using a known quantum yield ^6, 8^. Actinometry, which is useful for measuring light, does not provide inputs to help optimize the light source, as the measured data only signifies the cumulative number of photons absorbed by the chemicals used, as it is exposed to light over a period of time.

As discussed above, experimental techniques have worked well in air media; however, in water media, operators face multiple operational challenges ^9^. To overcome these challenges, simulation techniques have been employed to understand the radiant intensity in a water medium. Simulation techniques, such as discrete ordinates, are models in a virtual environment that help understand theoretical light irradiations acting at the point of interest.

Over the years, two main simulation approaches have been used in the literature: Eulerian (volumetric reaction rate-based) and Eulerian-Lagrangian (particle tracking-based) frameworks ^10– 13^. Several models have been proposed for radiation distribution and evaluation of the kinetic rate constants of microbial inactivation ^14– 17^. It has been shown that reactor dynamics, radiation, and kinetics can be solved using simulation packages, such as COMSOL (RRID:SCR_014767), ANSYS (RRID:SCR_022135), or OpenFOAM, based on Computational Fluid Dynamics (CFD) for reactor modeling. Within the Eulerian framework, the conservation equations for mass and momentum are solved. A comparison of the average particle (in this case, simulated microorganisms) values helped determine the overall performance of the UV reactor ^18^. Within the Lagrangian simulation framework, the trajectories of microorganisms (considered dispersed particles) are computed by considering the Newtonian equation of motion ^18^. The inactivation is determined using the accumulated light energy within the described path ^18^. Unluturk *et al.* 2004 ^19^ and Wright *et al.* ^11^ used a combined Eulerian-Lagrangian framework to simulate UV photoreactors for microbial water disinfection.

Keshavarzfathy *et al.* ^20^ elaborated on the need to conduct studies on design concepts that lead to a better understanding of the hydrodynamic interactions and reactor performance. This research studied the development of a model for the simulation of UV light emitting diode (LED)-based reactors in the Eulerian framework. The Monte Carlo method is another approach that has been considered in literature ^21, 22^. This approach is a stochastic method that allows for flexible geometry and adapts well to complex statistical simulations ^23^. This technique involves tracking the trajectory of a large number of photons and computing the location where they are absorbed in a 3-dimensional space. Busciglio *et al.* ^24^ further considered a probabilistic approach to radiant field modeling in a system, and validated the model using Monte Carlo simulations. The research found significant agreement between the two techniques. The approaches discussed above can cover all domains within the reactor if sufficient particles are considered for analysis. Nevertheless, the subject of radiation modeling and transfer in different types of media has been approached from multiple directions in the literature, and multiple challenges have been observed. Shah *et al.* reviewed two methodologies for modeling: SURF (simultaneous UV fluence rate and fluid dynamics) and TURF (three-step UV fluence rate and fluid dynamics), and concluded that the CFD models can predict the dosage received by water better than applying the average dosage to the system based on the power of lamps ^25^. The performance of a reactor depends on multiple factors within the system, including the interaction between radiation type, radiation dynamics, and reactor design ^26^. In a 3-dimensional domain, obtaining an accurate prediction of the radiant field and intensity reaching a point of interest requires powerful computing capability and space.

Although the aforementioned simulation and experimental techniques are used for their respective applications, these models and tools also lack some inputs that are necessary for the accurate and valid prediction of light irradiation in a flow-based system owing to the inability to incorporate certain parameters that play a key role in the amount of irradiation reaching the point of interest, including i) accurate source modeling to accommodate the radiation pattern of the light source; ii) scattering and reflections incurred owing to the design of the emission system and turbulent flow of water through the system; and iii) inputs on how to optimize the light reaching the point of interest given that UV LEDs operate at low efficiencies. However, the exact knowledge for optimizing the amount of light reaching the point of interest remains unknown in most cases, and these parameters have been continuously evolving over time. Light sources have moved from mercury lamps to LEDs, which are much smaller than the former, making recent germicidal systems less bulky.

In this study, a method for predicting the radiant intensity reaching a point of interest within a water-based medium is studied using optical ray tracing by considering the actual radiation profile of the selected UV LED, optical phenomena occurring within the medium, design optimization, and changes in intensity at interfaces to overcome challenges faced in both experimental and simulation techniques currently used. The ray tracing technique is mainly used in the pre-production stages of light-source manufacturing. It has been widely debated how effective ray tracing is compared with the traditionally used Monte Carlo algorithms. Li *et al.* ^27^ compared the two techniques for spine lesions and observed that ray tracing significantly overestimated the volume of target covered by the dose for one case but saw that the estimated dose difference was within 3% between the two techniques for the three other cases studied. The main difference in the former case was the presence of multiple air cavities. In conclusion, the authors mention that the ray tracing technique is “adequate” for use in most cases, but it would need to be validated with other techniques for further use of simulated data ^27^. In a similar study comparing ray tracing with the reverse Monte Carlo method for application to a GEO orbit, Benacquista *et al.* concluded that the ray tracing method is fast, but presents intrinsic limitations that need to be verified before further use ^28^. Monte Carlo simulations have been used in the literature for reactor modeling and have been proven to be time-consuming and require a large computational space ^29– 31^.

To the author’s knowledge, this study is the first to employ ray tracing in a germicidal system and attempt to trace the path of ultraviolet light as it propagates through the system and water medium. There are multiple tools from different manufacturers that employ ray tracing theory, such as 3Delight ^32^, POV-Ray ^33^, and ZeMax ^34^. ZeMax Optic Studio was used to design and simulate the system in this study. Given the limitations seen in the literature, this study analyzes the validity of the method by comparing it with existing techniques employed in academia and the industry. This study conducts a step-wise analysis of the designed system by comparison with radiometry in an air medium and uses the obtained data to quantify the effect of a quartz tube on irradiation in air. This quantification provides insight into the amount of light lost at multiple working distances using this method. Discrete ordinate method (DOM) simulations, commonly employed in photoreactor simulations ^35– 38^ have been used to understand the steps and challenges between the two simulation techniques. The study also develops a model to simulate the presence of water and compares it with a lab-based method used to calculate the number of photons entering water i.e., ferrioxalate actinometry, and compares the increase in radiant intensity as the light passes through the setup. Finally, a model was built to provide insight into the radiant energy distribution within a complex system of four wavelengths and an understanding of how the light intensity changes as it propagates within the tube.

## Methods

### LEDs source, fixture design and spectral characterization

**Figure 1.**
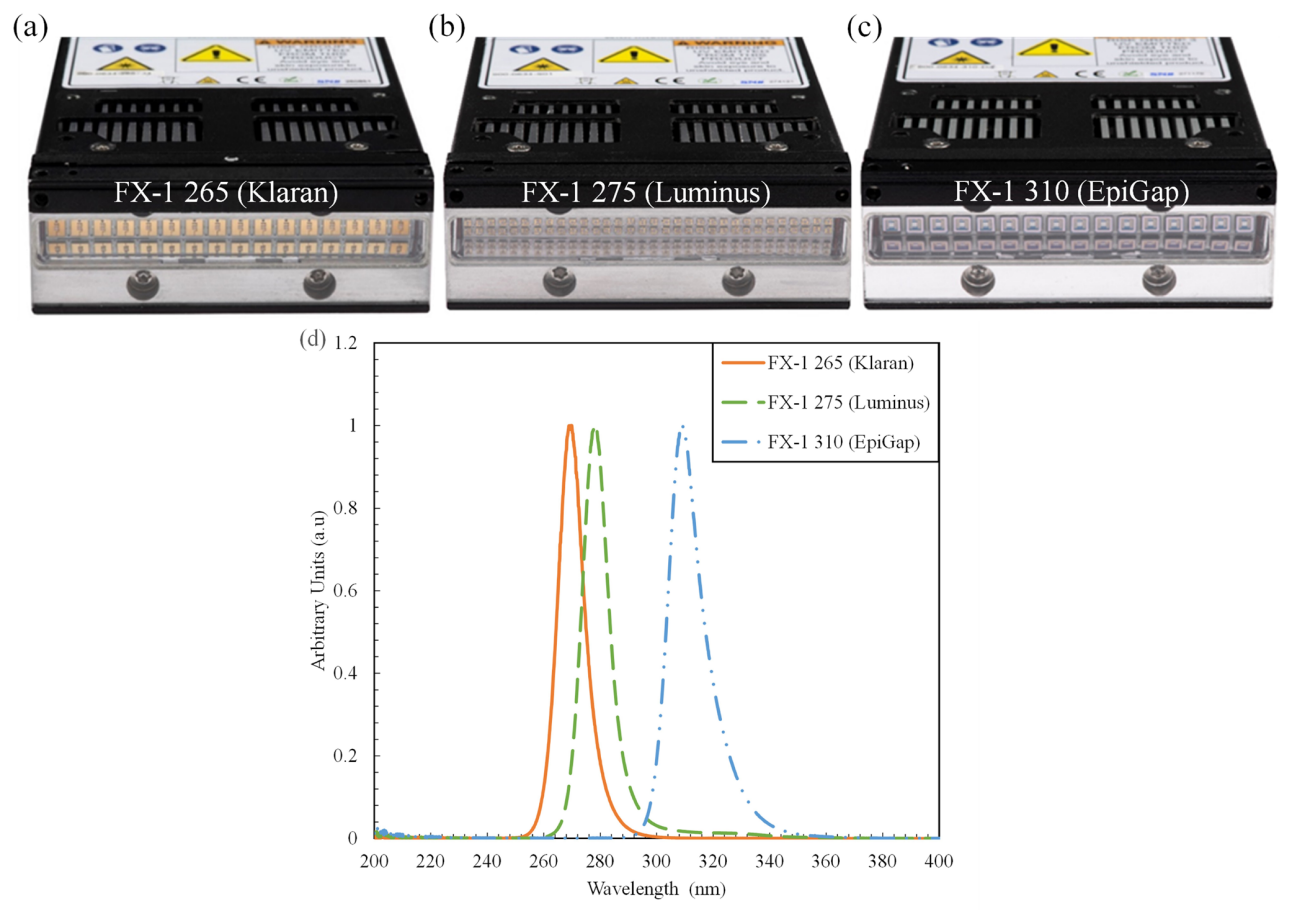
( **a**– **c**) FX-1 sources and ( **d**) Relative spectral intensity for each light source as recorded by ILT RAA4 spectroradiometer.

Three LED light sources with spectral emission in the UV-B and UV-C ranges were selected for this study. The LEDs chosen for this work were 265 nm (KL265-50U-SM-WD, 70mW, Klaran), 275 nm (XBT-1313-UV-A150-AG270-00, 8mW, Luminus), and 310 nm (EOLS-310-697, 50mW, EpiGap) as part of the REWATERGY project (Project No. 812574). To conduct the analysis and experiments, the LEDs were built onto the COBRA Clean FX-1 (hereafter called FX-1) supplied by ProPhotonix (See Figure 1 a-c). Spectral measurements were made to ensure that the emission of the LEDs was within the scope of this study using a spectroradiometer (2003357U1, International Light Technologies) with RAA4 coupling optics. Figure 1d shows the spectrum of each LED relative to its peak wavelength.

The FX-1 series by ProPhotonix was used to test the LEDs selected for this study ^39^. The device (emitting window size of 76.8 mm X 28 mm) was designed to fit the UV LEDs chosen according to their respective footprints. The FX-1 265 and FX-1 310 accommodated 16 LEDs each ( Figure 1a, c), while due to the size of the 275 nm LEDs (1.35 mm *1.35 mm), 64 LEDs were accommodated on FX-1 275 ( Figure 1b). Light is emitted and controlled by a controlled conditioner driver that uses a 48V DC safe current. The driver has a microcontroller on the printed circuit board (PCB), which monitors the temperature of the substrate and applies cooling via fans on the device. The driver helps maintain a safe electric current to avoid shorting or burning out the LEDs. It uses a 0-10 volts analogue signal corresponds to a 0-100 percent intensity range.

**Figure 2.**
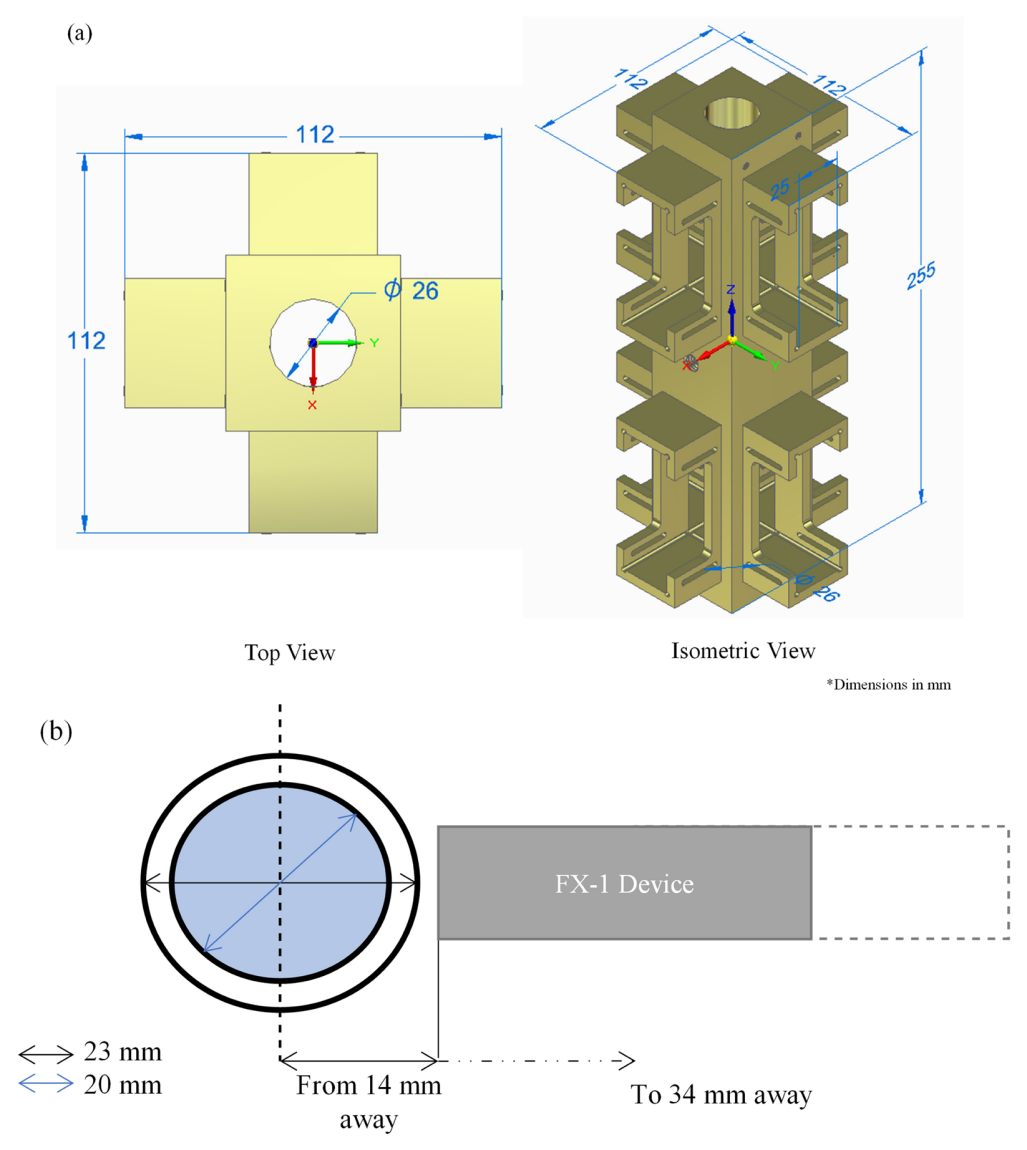
( **a**) UV Fixture and ( **b**) Representation of distance from the center of the tube to the source window.

To conduct actinometry and further tests, a custom designed UV fixture was manufactured (See Figure 2a). The UV fixture can accommodate up to 8 FX-1’s and a quartz tube. The quartz tube can be connected to the sampling tank and outlet to enable a flow through system. The quartz tube used for this study has an external diameter of 23 mm, an inner diameter of 20 mm and a length of 100 mm (FAB028553, Multi-Lab Ltd). The devices, when mounted on the UV fixture, can be moved to multiple working distances (from 14 mm to 34 mm away from the center of the quartz tube – see Figure 2b representation). The UV fixture, seen in Figure 2a, is made up of aluminum material to ensure any light lost can be reflected back into the quartz tube.

**Figure 3.**
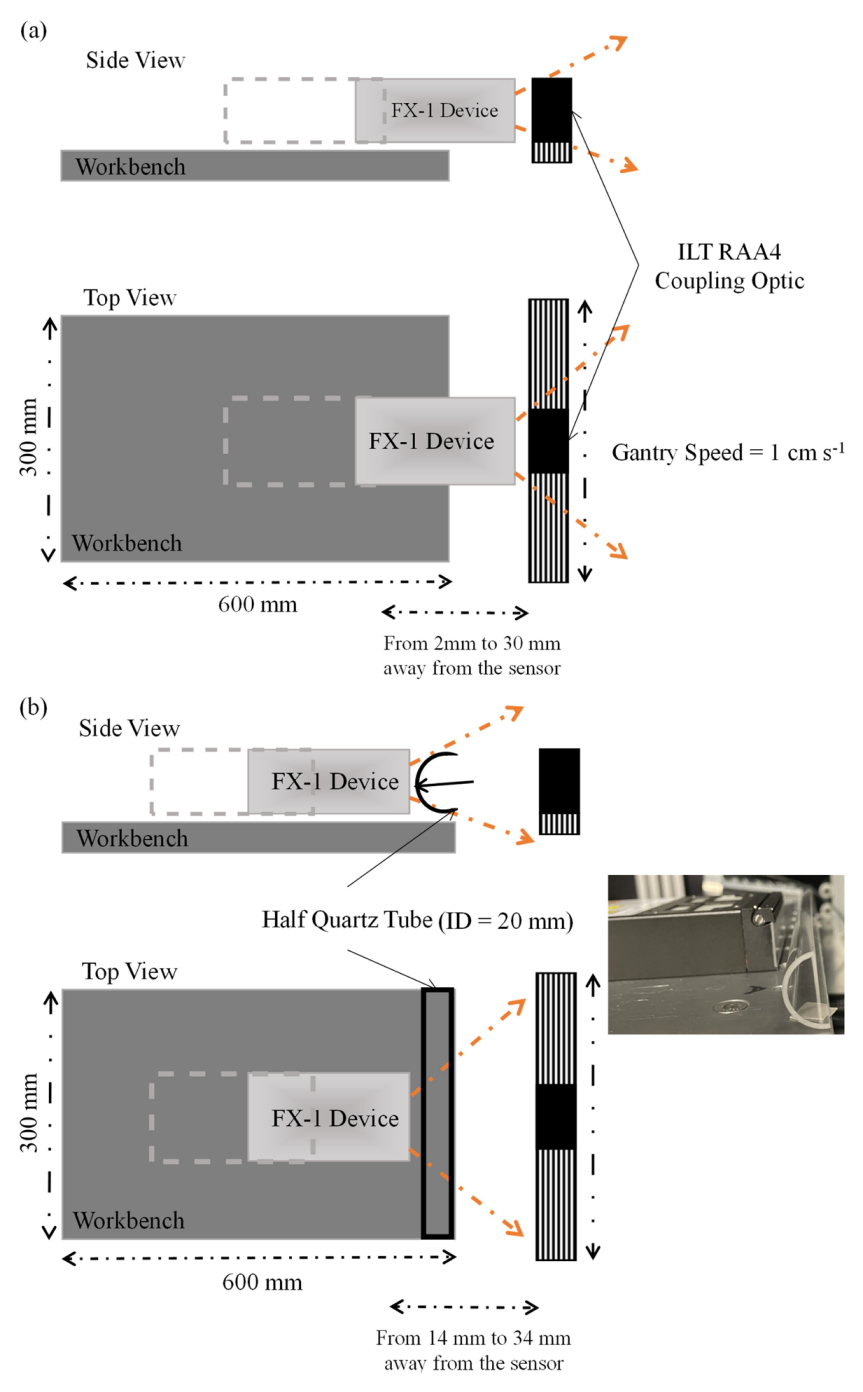
( **a**) Representation of X-Y tester for radiometry, ( **b**) Representation of X-Y tester for half quartz tube radiometry (Not to scale).

To characterize and quantify the intensity of light emitted from the device, a spectroradiometer has been used. The spectroradiometer is the ILT 950UV series measuring radiation in the range of 210 nm to 1100 nm ^40, 41^. To understand the complete emission profile of the device and other characteristics, an X-Y based motor gantry tester was used ( Figure 3a). The tester consists of a workbench (where the device can be mounted and moved to multiple working distances away from the optical sensor) and a motor gantry which supports the optical sensor and moves as required in longitudinal and lateral directions. The entire set-up is controlled using a LabVIEW VI that has been set-up and programmed to collect data from measurements for further analysis. The entire set-up is enclosed in a black box to avoid exposure to harmful UV radiation.

### Set-up to quantify the effect of quartz tube

One of the objectives of this study was to quantify the effect of quartz tubes and compare it with the results obtained from the ray-tracing model. To achieve this, a custom-manufactured half-quartz tube cut along its length was used. The tube was from Multi-Lab Ltd. (FAB027469, 23 mm outer diameter, 20 mm inner diameter, and 100 mm length). Figure 3b shows a schematic representation of the change in setup for this experiment, and Table 1 summarizes the quartz tube properties, including the transmittance data obtained from the manufacturer. The quartz tube and device were placed according to the dimensions of the UV fixture discussed above ( Figure 2a) and tested at the same working distances as those available on the fixture.

**Table 1.** Material and optical properties of the quartz tube.

Property	Typical Value
**Density (ρ)**	2.2 × 10 ^3^ kg m ^-3^
**Refractive Index**	1.485
**Transmittance**	At 265 nm - ≈0.89 At 275 nm - ≈0.90 At 310 nm - ≈0.92

### Optical modeling of the devices with ray tracing

ZeMax Optic Studio (Version 19.4, proprietary software) was used for the optical simulation. An alternative to this software is the Ansys Optics 2023 R2 ^42^. This tool is commonly used by device manufacturers in the early stages of device production, as it helps understand the ray paths, predicts the theoretical peak intensity (according to the LED datasheet optical power output), and provides inputs on ways to optimize the use of reflectors and/or optical components within the device. The tool employs a low-cost and quick technique of ray tracing. The software models the propagation of light from the designed source, through the system, and on to the final point of interest. The resulting distribution of rays within the system is used to predict a wide range of light parameters according to the operator’s interest ^34^.

### Governing equation

Ray tracing involves the use of two fundamental properties: ray position and direction. The position and direction of the ray in the Cartesian coordinate system are defined in Equation 1 and Equation 2.


$$\;\vec{r}=\{x,y,z\}\;(1)$$

$$\;\hat{k}=\{l,m,n\}\;(2)$$

In Equation 1, $\vec{r}$ is the position of the ray and ( *x, y, z*) are the coordinates measured in units of length, depending on the type of system analysis. In Equation 2, $\hat{k}$ is the direction of the ray, and ( *l, m, n*) are the direction cosines of the unit vector that points along the ray. Both quantities are measured based on local or global coordinates relative to the reference frame input by the operator. If a ray is propagated by distance *x*, where *x* is the length in SI units, the new coordinates of the ray are given by Equation 3.


$$\;\vec{r^{\prime }}=\vec{r}+x\hat{k}\;(3)$$

To predict the ray path by refraction, reflection, or diffraction within the setup, the tool employs Snell’s law in vector form at the point of intersection with a surface ( Equation 4)

$$\;n^{\prime }(\hat{N}*\hat{k}\prime )=n(\hat{N}*\hat{k})\;(4)$$

Where *N* is the unit normal vector of the surface at the point of contact and *k* is the ray direction cosine vector. Equation 4 changes with the kind and type of optical phenomenon detected at the point of intersection between the surface and ray. To optimize and understand the associated ray path and light system, the tool has the option for two types of ray tracing: sequential and nonsequential ray tracing. In the sequential mode, light rays are limited to propagating from one point to the next and are not flexible for complex systems. Nonsequential ray tracing allows rays to propagate through the components within the system and allows ray splitting, scattering, and reflections to occur during simulation. This method of ray tracing means that there is no specific sequence for the movement of rays within the system, that is, the rays may hit any part of the designed system and move in any direction based on the optical phenomena detected at the point of contact by the software ^34^.

### Boundary conditions and material properties

The non-sequential mode of ray tracing was employed in this study. Contrary to the case of DOM simulations, the media between the LED array and the radiometer need not be included in the simulation to calculate radiation transport. In the case of ZeMax, the body is designed using shapes available on the software, such as point sources, ray sources, and two-angle sources ^34^. In this study, the source was designed as a radial source, and the other parts were designed using multiple shape options available on the tool, based on the device used to match and replicate the actual manufactured device. Each LED footprint was considered when designing the device on the tool. Dimensions such as the thickness of the LED package, size of the actual light-emitting surface, and optical power output from the source were drawn from the datasheet, while other parts were designed according to the dimensions provided by ProPhotonix. A screengrab of the modeling stage on the optic studio can be seen in the Additional Data (Figure S1) ^43^.

In the ZeMax user interface, the entire emission radiation pattern can be input into the source properties, ensuring that the simulated source irradiation is similar to the manufactured light source. The radiation pattern was extracted from the datasheet of the LEDs and used as an input to the tool. The 265 nm and 310 nm LEDs have viewing angles of 120° and 130°, while the 275 nm LEDs have a higher viewing angle of 150°.

Optical power is a key parameter that controls the simulated values. First set of simulations were conducted for each wavelength using the power output of the LED (mW) specified in the LED manufacturer datasheet to understand the theoretical intensities delivered to the points of interest. Using radiometric experiments conducted in air, the actual power output of the LEDs was recalculated and used as input optical power for the second set of simulations. This helps to ensure that the model behaves closely to the actual measurements. This provides an understanding of how the LED behaves within the device by controlling the light output. Once this is completed, the model can be further used to simulate the presence of a quartz tube and water in front of the source.

**Figure 4.**
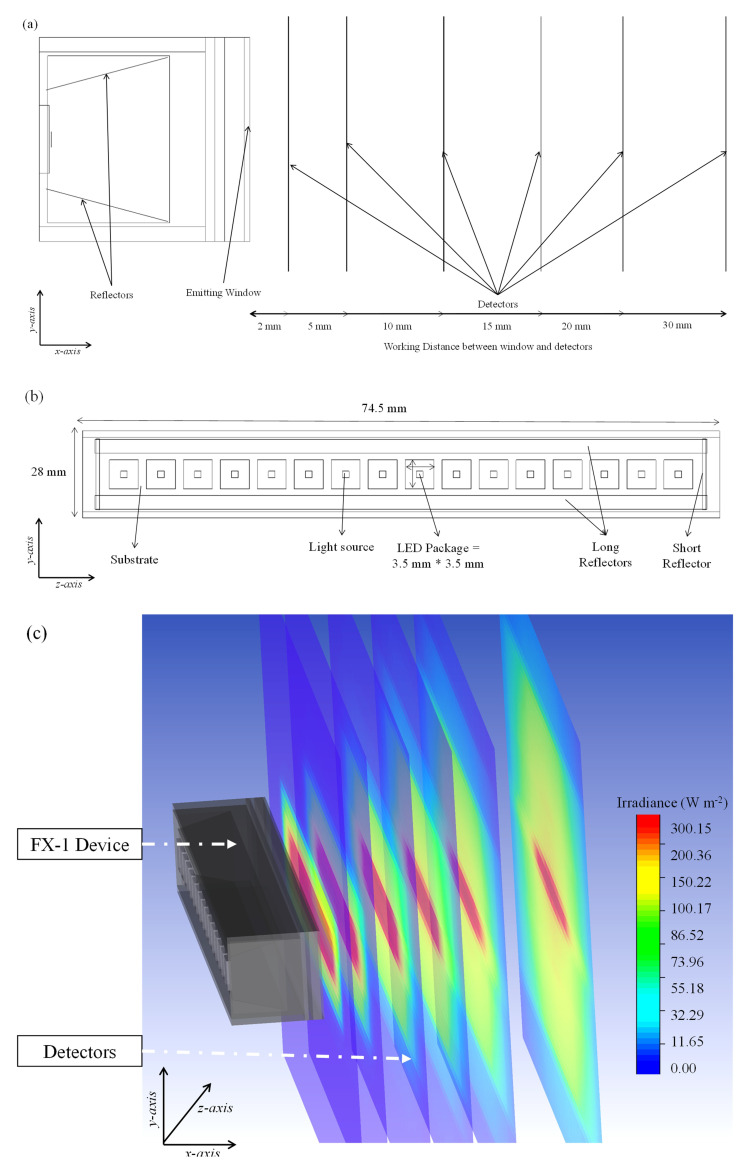
( **a**) Side view of the designed model on ZeMax interface, ( **b**) Top view of the designed model ( **c**) Section view of simulated FX-1 265 Optical Model.

A quartz tube is not readily available on the interface, and multiple object types were considered before arriving at the use of a cylindrical object type. The quartz tube was modeled using a Boolean technique to simulate the hollow tube, and the inner material of the pipe was changed to “ *water*” for water medium simulations while the outer cylinder material was changed to “ *quartz*.” The properties of the quartz tube were based on the properties of the quartz library-loaded material on the tool. The properties of the tube were as per the data derived from Bass *et al.* ^44^. On the tool, the main parameter that dictates the simulation accuracy is the number of rays within the simulation. For a rough understanding of the light emission and path within the system, a low ray count will work; however, for accurate simulations, a higher ray count is recommended. In this study, all simulations were conducted at 10 ^6^ rays per simulated model. To quantify and analyze the data from light simulations, detector rectangles (here onwards called analytical detectors) were designed within the model. Analytical detectors were created at multiple working distances to simulate and quantify the effect of the working distance on the intensity of light from the source. Figure 4a, b shows a sectional view of the 265 nm source designed on the software depicting the light source, the mechanics within the device, and the detector objects as black lines in the *x* direction. For simulations involving the generation of a water medium, the refractive index was changed for each simulated wavelength. The input data for these models are listed in Table 2.

**Table 2.** Wavelength specific refractive index and quantum yield in water ^45, 46^.

Wavelength	Refractive index	Quantum yield (ɸ)
**265 nm**	1.3572 ^45^	1.2281 ^46^
**275 nm**	1.3540 ^45^	1.2629 ^46^
**310 nm**	1.3478 ^45^	1.2281 ^46^

### Characterization using chemical actinometry

Actinometry is a method by which the number of photons in a beam can be measured using a chemical system that absorbs incident radiation in a defined space of a reactor. This method integrally determines the number of photons with respect to time. The reactants used within this chemical system undergo a light-induced reaction in which the quantum yield is known. The quantum yield (Φ(λ)) of a photochemical reaction can be defined as the number of events, such as the molecules formed divided by the number of absorbed photons of that wavelength ^47^. The measurement of the reaction rate allows for the calculation of the absorbed photon flux ( Equation 5).


$$\;q_{p}\left(abs,\lambda \right)=q_{p}^{o}\left(\lambda \right)\left(1-10^{-A\left(\lambda \right)}\right)\;(5)$$

where $q_{p}^{o}$ is the incident photon flux and A (λ) is the decadic absorbance. Different chemical systems are listed by the International Union of Pure and Applied Chemistry (IUPAC), such as solid and micro heterogeneous-phase chemical actinometers, gas-phase chemical actinometers, and liquid-phase chemical actinometers. Based on the wavelengths studied and the type of setup used, a liquid-phase chemical actinometer was selected. Specifically, a potassium ferrioxalate (K _3_[Fe(C _2_O _4_) _3_].3H _2_O) based chemical system was used in this study. The chemical system is also recognized as the Hatchard-Parker actinometer by IUPAC and is widely accepted as a standard actinometry test for ultraviolet wavelengths. The actinometer had a wavelength range of 250 – 500 nm with a quantum yield (Φ) of 1.25 – 0.9 ^48^ *.* The quantum yield used in the calculations for each wavelength are listed in Table 2.

**Figure 5.**
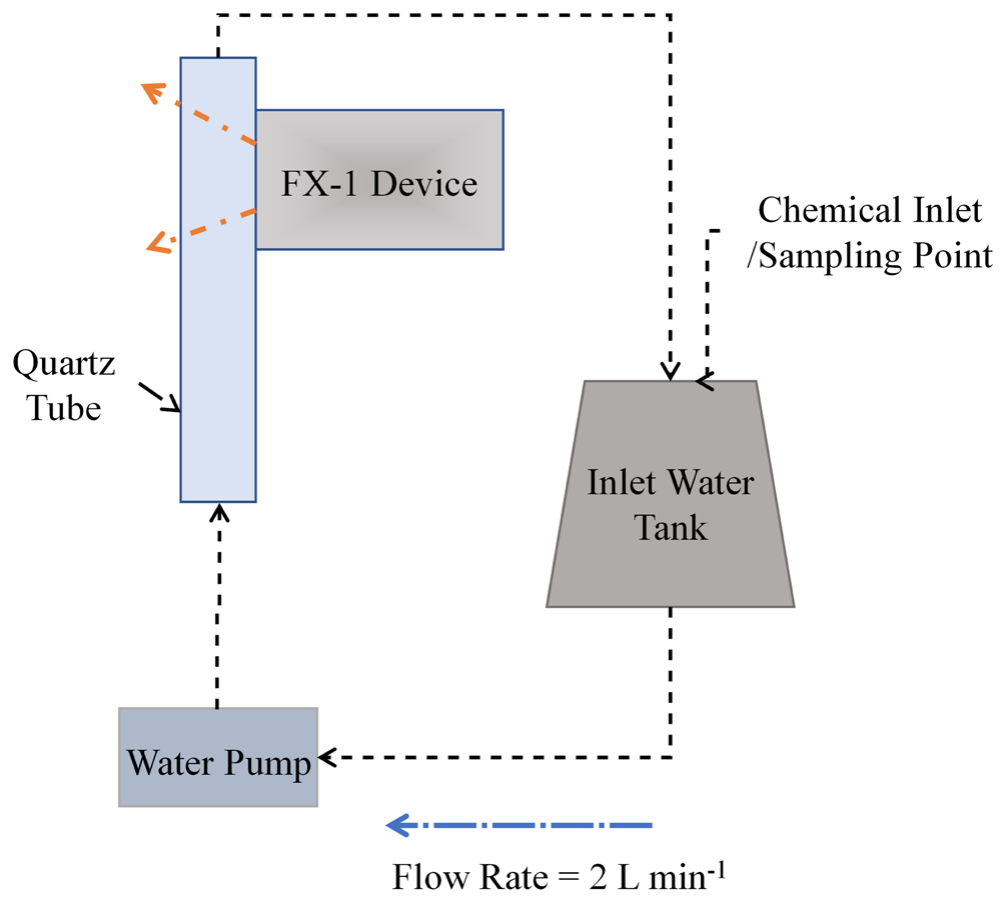
Schematic representation of the set-up used for actinometric experiments.

A UV fixture was used to characterize the device using chemical actinometry ( Figure 2a). For each test, only one device with a specific wavelength was used at a distance of 14 mm from the center of the quartz tube. A pump with a flow rate of 2 L min ^-1^ has been used to flow the prepared solution through the fixture. Figure 5 shows a schematic of the setup used. A 2 L MilliQ® water matrix consisting of Oxalic Acid (H _2_C _2_O _4_.2H _2_O, Scharlau), Ferrous Sulphate (Fe _2_(SO _4_) _3_.5H _2_O, ThermoScientific), 1 N Sulfuric Acid (H _2_SO _4_, Scharlau), 1,10 Phenanthroline (C _12_H _8_N _2_, Scharlau), was used as the base matrix for these experiments. The matrix was irradiated, cyclically, by each source for a maximum period of 50 minutes and samples were collected at specific intervals of time (See Additional Data, Figure S10 ^43, 49^). The experimental procedure was the same as that used by Hatchard *et al.* ^48^. The irradiated samples were collected and added to a buffer matrix consisting of water (H _2_O), 96% Sulfuric Acid and Acetic Acid sodium salt trihydrate (C _2_H _3_NaO _2_.3H _2_0, Scharlau) and rested for a minimum of 30 minutes. The absorbance of these samples were then measured at 510 nm to calculate the incident photon flux. Tests were conducted for each wavelength on three separate days, with samples extracted at different time intervals to ensure that repeatable and reproducible data were obtained.

### Discrete ordinate method (DOM) modeling

***Governing equation*.** DOM is one of the available models used to calculate the radiation transport implemented in Ansys Fluent (2021R2, RRID:SCR_022135) in the multiphysics ANSYS ^TM^ platform ^50^. This method solves the radiative transfer equation over a domain of discrete solid angles. In this technique, the incident radiation is calculated by integrating the radiant intensity along a spherical space. This method employs the discretization of spatial directions and solves the radiative transfer equation (RTE, Equation (6), 50) in each direction. It calculates radiation intensity as a function of absorption, scattering, reflection, and emission. This equation describes the conservation of radiative intensity in a direction of space.


$$\frac{dI_{\lambda ,\vec{\Omega }}}{ds}=\underset{Absorption}{\underset{︸}{-k_{\lambda }I_{\lambda ,\vec{\Omega }}}}\;\underset{\begin{array}{c} Out\\ Scattering \end{array}}{\underset{︸}{-\sigma_{\lambda }I_{\lambda ,\vec{\Omega }}}}\;\underset{\begin{array}{l} Thermal\\ Emission \end{array}}{\underset{︸}{+\frac{k_{\lambda }T^{4}}{\pi }}}\;\underset{InScattering}{\underset{︸}{+\frac{\sigma_{\lambda }}{4\pi }\int_{\Omega^{\prime }=4\pi }p\left(\vec{\Omega^{\prime }}\to \vec{\Omega }\right)I_{\lambda ,\vec{\Omega }}d\vec{\Omega^{\prime }}I_{\lambda ,\vec{\Omega }}}}\;(6)$$

where *I _λ, $\vec{\Omega }$_* is the intensity of photons with wavelength λ propagated along the direction $\vec{\Omega }$ ; k _λ_ is the volumetric absorption coefficient, σ _k_ is the volumetric scattering coefficient, σ is the Stefan-Boltzmann constant, T is the temperature in degrees Kelvin, and *p* ( $\vec{\Omega }\prime$ → $\vec{\Omega }$ ) is the phase function that describes the directional distribution of scattered radiation.

The device was designed using Solid Edge (V 221.00.03.003, RRID:SCR_021075)CAD software and converted to ANSYS ^TM^ Workbench for radiation simulations using Fluent. In this study, light was assumed to be monochromatic. Each LED within the device was simulated as a flat surface-emitting light. The following assumptions were made before conducting simulations: i) thermal emission is neglected by setting the temperature to 0 Kelvin; ii) density and viscosity are considered constant, as per standard material properties, for the wavelength range studied; iii) absorption coefficient of 0.00074 m ^-1^ and scattering coefficient of 0.00049 m ^-1^ have been assumed for air in the wavelength range (250 – 320 nm) from previous studies ^51^.

**Figure 6.**
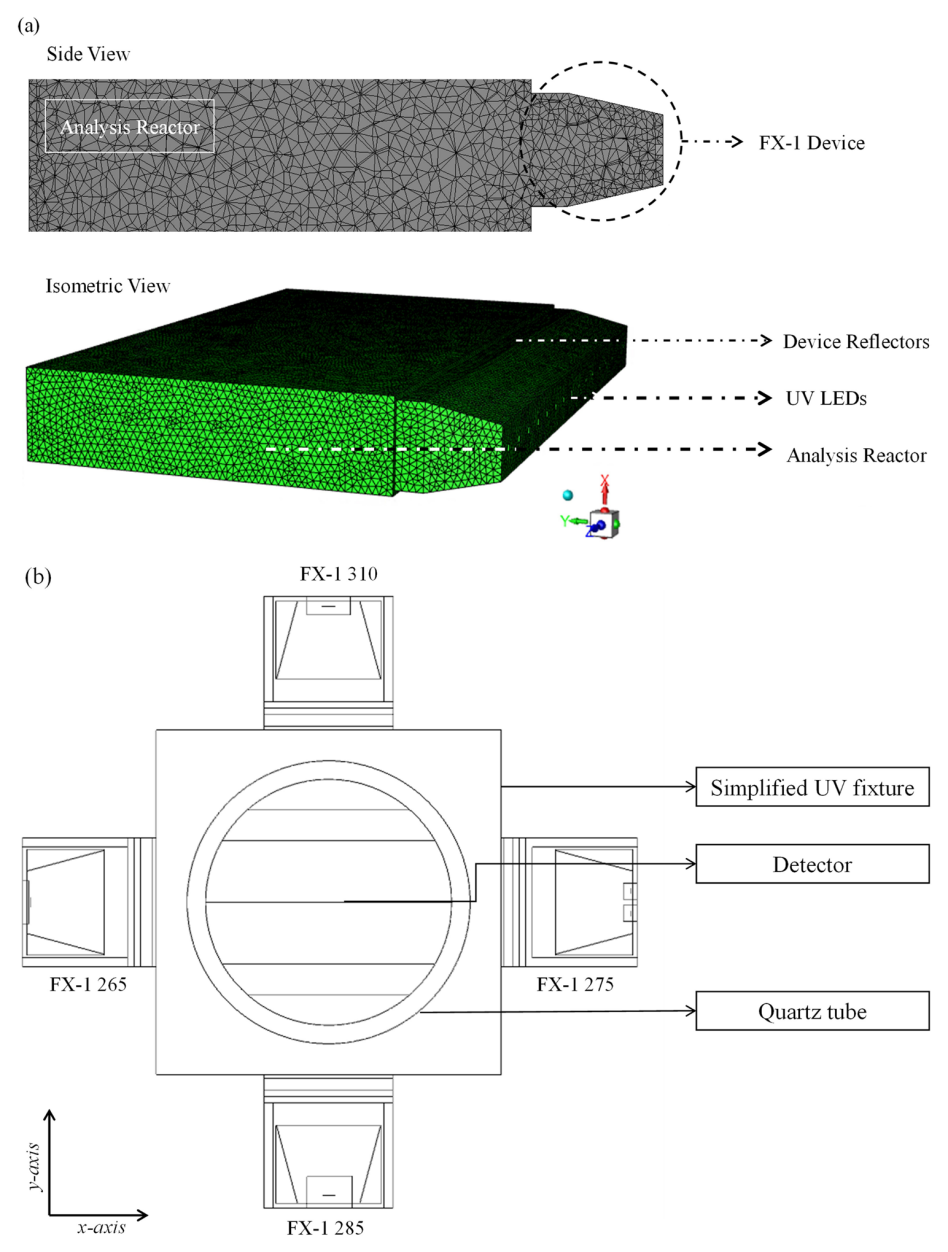
( **a**) Meshed DOM model of FX-1 265, modeled in ANSYS Fluent, ( **b**) Top view of the 4-wavelength complex system.

***Boundary conditions and material properties*.** To simplify simulation time and the number of equations within the model, only the emitting window section (light head) of the device has been modeled (see Figure 6a), with the same dimensions as the experimental system: 76.8 mm × 28 mm with 4 reflectors inside the emitting window. For each wavelength, the LED size and number of LEDs were modeled according to the supplier’s design and datasheet specifications. In this study, the reactor was considered to be a simple black box that absorbs any radiation irradiated by the source. The reactor had slightly larger dimensions than those of the emitting window. Before extracting data from the simulations, a mesh sensitivity analysis was conducted. For further information, please refer to the Additional Data (Table S1, Figure S2-S4 ^43, 49^).

For simulation on Fluent, the following conditions have been used.

a) Boundary Conditions: (i) light source – direct irradiation and beam width as per manufacturer specifications; (120° for 265 nm, 150° for 275 nm, and 130° for 310 nm FX-1); (ii) Reflectors –zero internal emissivity and diffuse fraction; (iii) Reactor, emitting window, and LED substrate –100% internal emissivity and diffuse fraction.
b) The refractive index of air has been accounted for at each wavelength from literature studies (265 nm – 1.00029777, 275 nm – 1.00029570 and 310 nm – 1.00029023 ^45^).
c) For the light source, an angular discretization of 15 × 15 solid angles per octant was used to capture the LED beam angle.
d) The optimum mesh size from the mesh sensitivity resulted in an average of 417,876 cells per wavelength using an inflation mesh at the LED surface and the surface mesh for the reactor body.
e) Second-order upwind solution method for the DOM model with up to 500 iterations to calculate the solution.
f) The convergence of the numerical solution was ensured by monitoring the scaled residuals to a criterion of at least 10 ^-6^ for discrete ordinates and energy variables.

### Modeling of a 4-wavelength germicidal system

Upon validation of the simulated model using the ray-tracing method, a complex system consisting of four FX-1 devices operating at different wavelengths was modeled in a water medium. The purpose of this model was to determine the contribution of each wavelength to the overall dose received by a water matrix. Therefore, to isolate each individual wavelength on the UV fixture, an additional UV source (FX-1 285) consisting of 16 LEDs irradiated at a peak wavelength of 285 nm (XST-3535-UV, 40mW, Luminus) was added to the model. The model was modified to replicate a UV fixture, as shown in Figure 2a. The design was simplified to a shorter simulation time. The same number of rays (10 ^6^) as mentioned earlier was used in the simulations. Figure 6b shows a model of the complex system.

## Results and discussions

### Ray tracing and radiometry

**Figure 7.**
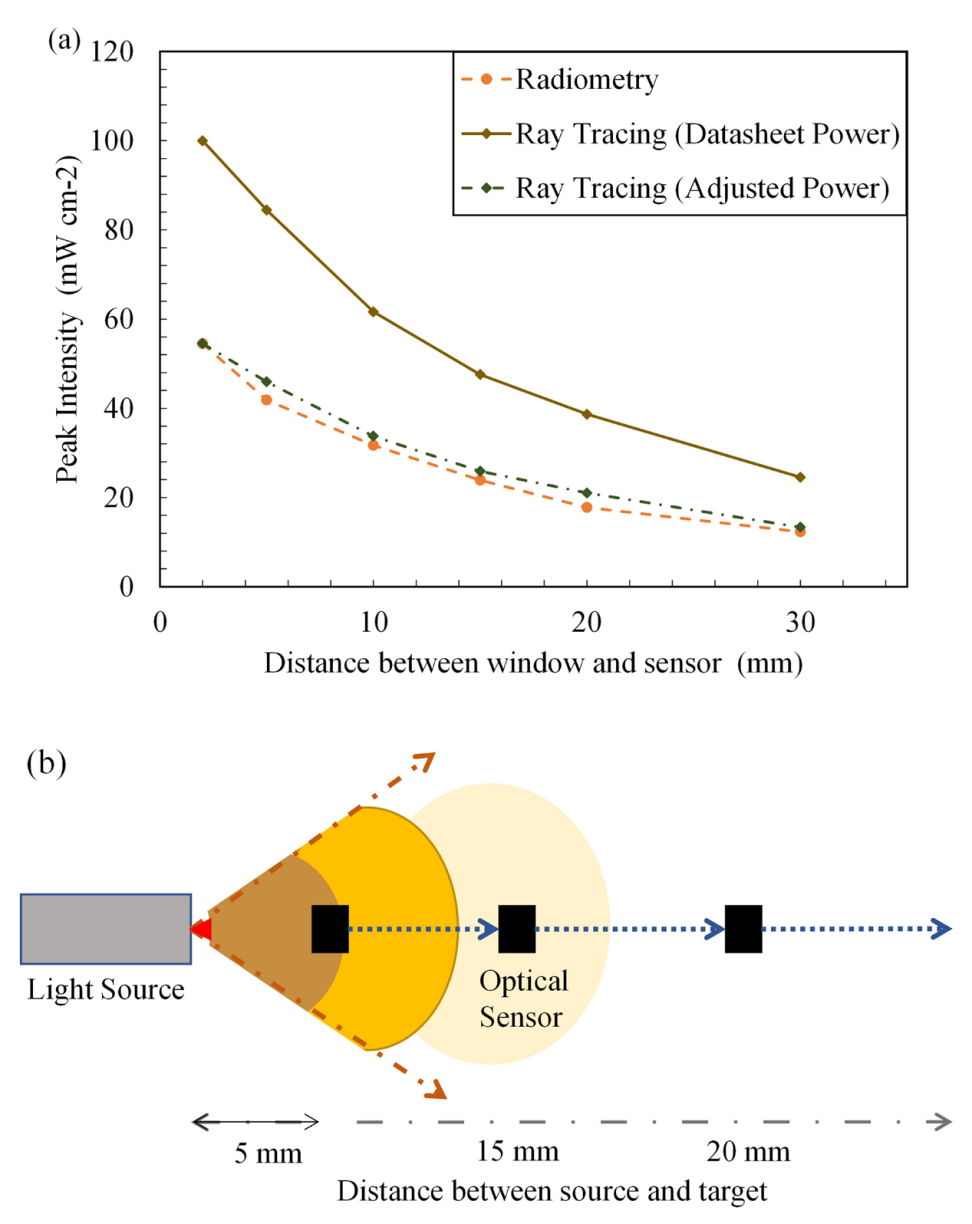
( **a**) Ray tracing data vs experimental data (FX-1 265), ( **b**) Representation of variance of light intensity with distance.

As mentioned earlier, one of the key inputs into the software is the optical power output per designed LED source. To understand how the tool is used in industrial applications, initial simulations were conducted using the data sheet mentioned in the optical power output ( mW) of the source. It can be seen in Figure 7a (diamond marker full line), the predicted peak intensity, at multiple working distances. The result predicted by the simulation is higher than the actual peak intensity delivered by the device. The manufacturer mentions that the 265 nm LED and 275 nm LED have an optical power output of 70 mW (at 500 mA) and 8 mW (at 40 mA), respectively, while the 310 nm LED has an optical power output of 50 mW (at 350 mA).

The LED manufacturer conducts tests, on the LED, in a near ideal environment and hence the theoretical peak intensity observed is considerably higher than the actual power output from the device. The actual power output from each LED must be dealt with on a case-by-case basis depending on the system and electronics that are driving the light source. LED device manufacturers conduct a rollover test that helps conclude the safe working current and, subsequently, the safe operating power output for higher lifetimes of the device ^52^. The test involves measuring the optical power output from the device with an increase in LED input current until the maximum input current capacity of the LEDs within the device. After a certain input current, any further increase does not result in a significant increase in optical power output. At this stage, any further increase means that the LEDs are generating more heat than light signal, hence are prone to degrade faster. Lighting device manufacturers call this point the rollover point and program the device input current to slightly lower or higher level (depending on safety of other device electronics) for safe operation and long lifetime of the LEDs. In the case of the FX-1’s used, upon rollover tests (refer to Additional Data for data on rollover tests, Figure S5 ^43, 49^), the operating current for the devices was set to 317 mA for FX-1 265, 139 mA for FX-1 275 and 437 mA for FX-1 310.

For example, the FX-1 265 rollover test results indicated an LED safe current for the device of 317 mA and a corresponding optical power output from each LED of 33 mW. Using this value as the input required for the source, the input power on the software was adjusted to check whether the model behaved similarly to the experiments. As shown in Figure 7a, the data from the adjusted power simulation (diamond marker dotted line) were very close to the data measured by the spectroradiometer (circle marker broken line) (± 5%). Data on the comparison between simulations for other wavelengths can be seen in the Additional Data (Figure S6 ^43, 49^). By comparing the two techniques, it was concluded that the device-based optics designed in the model were very similar to the actual device conditions. The base model of the light source can now be used to simulate other conditions and can be compared with these techniques.

For any light source, it is understood that the total intensity acting on a target area is a function of the light-emitting source and the distance between the source and target ^53^. With an increase in the distance from the target, the light intensity decreases as the spread of light is wider. The same number of photons emitted by the light-emitting surface is spread over a wider area at a longer distance from the source. Figure 7b depicts how light intensity decreases with increasing distance from the source. To characterize the light source using a spectroradiometer, working distances of 14–34 mm from the source window (in 5 mm steps), as designed within the UV fixture, were selected for measurements. The selected working distances provided an understanding of the behavior of light in the air within the fixture. Radiometric measurements were conducted on three separate days to ensure repeatability and accuracy of the obtained data. The experimental error obtained from the measurements was low and was found to be greater than the third decimal point. The error was calculated as the standard deviation of all measurements conducted for each wavelength. Although all wavelengths were measured and tested, only FX-1 265 data were presented, as the other wavelengths showed similar behavior and were in close agreement with the ray-tracing simulation data.

**Figure 8.**
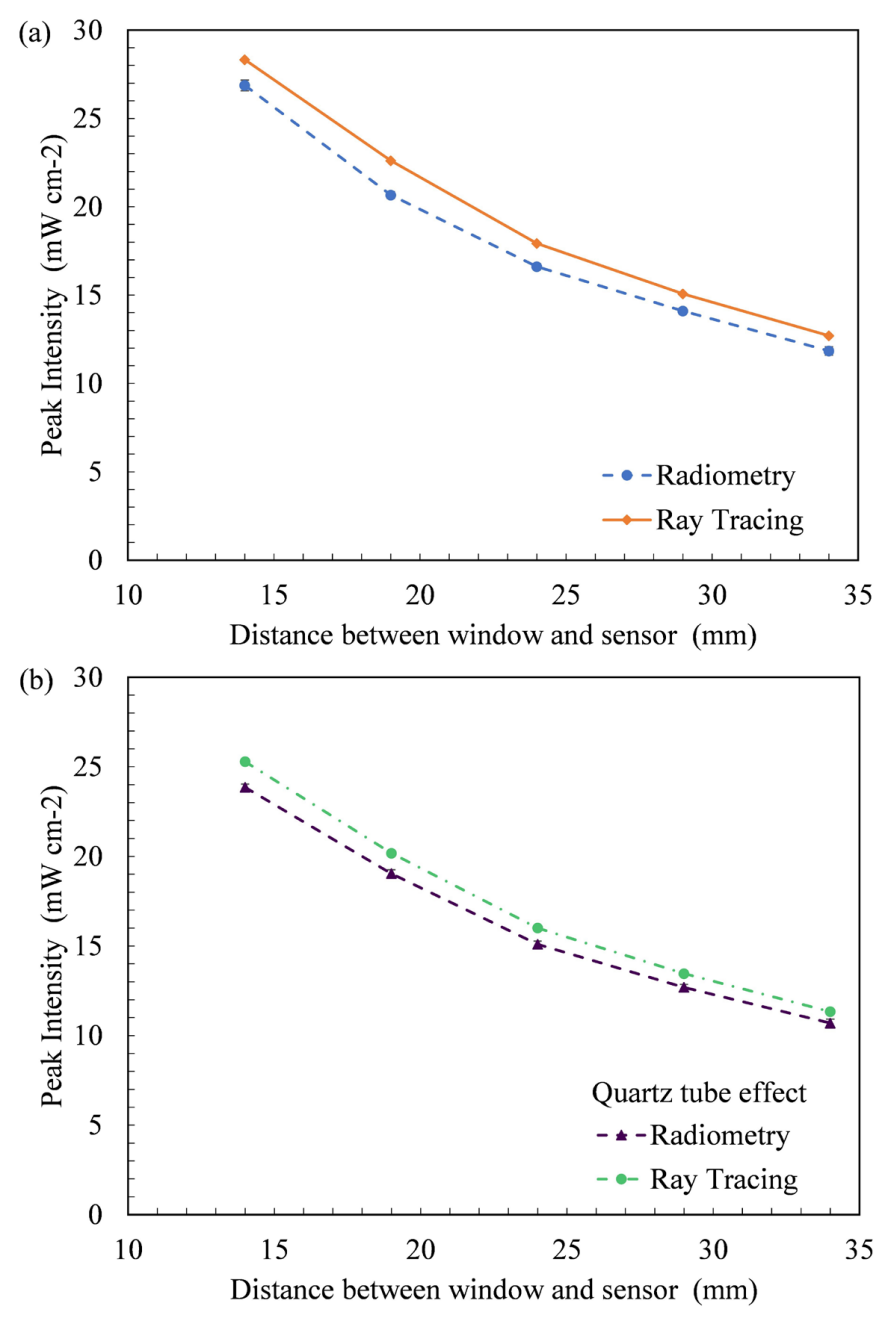
( **a**) Plot of peak intensity vs working distance (in air) and ( **b**) Comparison between radiometry and ray tracing in the presence of a quartz tube in front of the light source for FX-1 265.

Figure 8a plots the peak intensity measured at each working distance versus the working distance between the source emitting window and coupling optic for FX-1 265. It was seen that all the 3 wavelengths selected for this study follow the expected trend of a decrease in light intensity with an increase in working distance for both techniques (Figure S6, S7). Ray tracing simulations can be seen to have the same trend as the radiometric measurements with an average of ±7% difference between the two techniques for 265 nm source, while an average difference of 7% was observed for other two wavelengths. Similar to findings in the literature, the ray tracing technique overestimates the actual light intensity in the simulations. The difference and higher peak intensity observed can be attributed to the fact that in radiometric measurements, light bouncing off the workbench surface is lost while this does not happen in the simulation environment. Although designed as per dimensions and specifications, the design models do not fully replicate the actual environment and hence a difference is expected. Within the X-Y tester, the sensor/coupling optic moves in X-Y direction for a period of time and captures data at each set point and displays light measured at each point at that instant of time which could be another reason to explain the difference between techniques. To summarize the comparison between radiometric measurements conducted in air and the optical ray tracing simulations, Table 3 provides data on measured peak intensities at each working distance for the data seen in Figure 8a. Plots of peak intensity versus working distance for FX-1 275 and FX-1 310 can be found in Additional Data (Figure S6 and Table S2 ^43, 49^). Among the three wavelengths under study, relative to the FX-1 265, the FX-1 275 emitted 1.4% lower intensity while FX-1 310 emitted 23% higher intensity at 14 mm working distance away from the center of the system.

**Table 3.** Recorded peak intensity at multiple working distances using radiometry and ray tracing.

Working distance (mm)	Peak intensity (FX-1 265) (mW cm ^-2^)			
	Absence of quartz tube		Presence of quartz tube	
	Radiometry	Ray tracing	Radiometry	Ray tracing
**14**	26.87 ± 0.30	28.32	23.85 ± 0.44	25.28
**19**	20.65 ± 0.19	22.60	19.04 ± 0.26	20.18
**24**	16.61 ± 0.16	17.92	15.09 ± 0.22	16.00
**29**	14.09 ± 0.11	15.07	12.69 ± 0.42	13.45
**34**	11.84 ± 0.23	12.69	10.69 ± 0.34	11.33

### Effect of quartz tube

To analyze the impact of the quartz tube on light irradiated, Figure 8a shows a comparison between the radiometry measurements performed in the presence (circle marker broken line) and absence of the quartz tube at different distances from the light source (diamond marker full line) for FX-1 265. The quartz tube reduces the amount of irradiation reaching the target surface by 11% at a working distance of 14 mm from the center of the quartz tube and follows the same trend as expected. In the same way, the ray tracing simulations resulted in data similar to that of the radiometric measurements, as seen in Figure 8b (triangle marker line). Table 3 summarizes the measured and simulated data from these experiments and can be compared with the data measured in the absence of a quartz tube to understand the impact of the quartz material on the light irradiated for FX-1 265. In the presence of a quartz tube in front of the source, good agreement was observed between the two techniques within ±6% of the radiometric measurements for all wavelengths studied. Plots of the peak intensity versus the working distance for FX-1 275 and FX-1 310 can be found in the Additional Data (Figure S7 and Table S3 ^43, 49^). Compared to FX-1 265, FX-1 275 emitted nearly the same intensity (a difference of approximately 0.004%), while FX-1 310 emitted 32% higher intensity at a working distance of 14 mm away from the center of the quartz tube.

As mentioned earlier, this study attempts to analyze and quantify the effect of quartz tubes or quartz materials on the emitted light. This study employs a custom-manufactured quartz tube cut along its length to understand its impact. The tests provided an understanding of the impact of using quartz material in front of the light source and an input for the simulations. To better understand the effect of quartz, the manufacturer of the half-quartz tube provided the transmission curve of the quartz material used by the tube (see Additional Data Figure S8 ^43, 49^). The measurements were conducted, and the data were validated to ensure that the loss due to quartz material was well within the range expected according to the material specifications.

**Figure 9.**
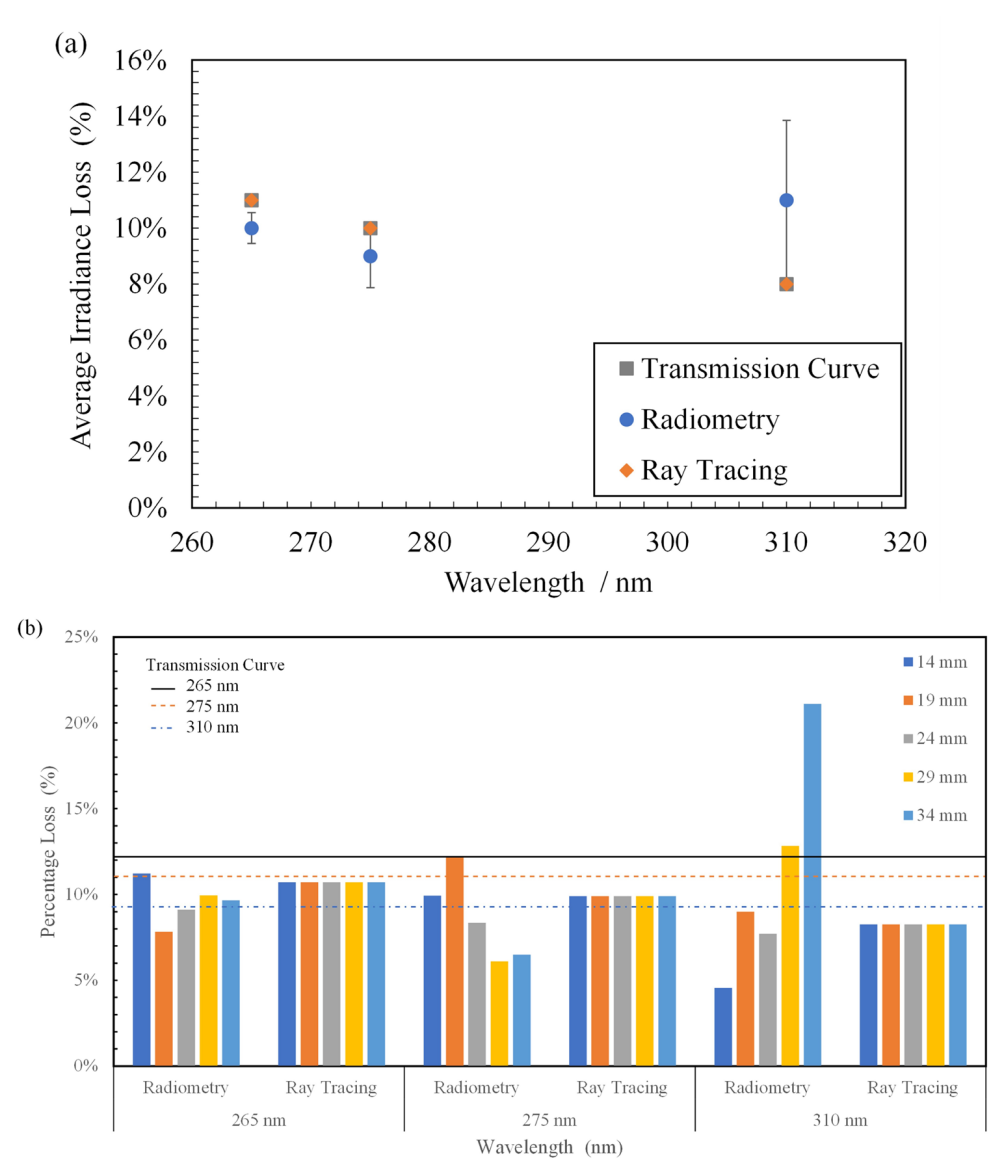
( **a**) Comparison between average light loss at multiple distances in radiometry, ray tracing and transmission curve due to quartz material and ( **b**) Plot of percentage light loss for all wavelengths at multiple working distances due to quartz material.

In Figure 9a, b, the average of all losses observed at multiple working distances in the radiometric measurements was compared with the ray tracing simulations and the transmission curve obtained from the manufacturer. While the data for FX-1 265 and FX-1 275 are in good agreement between the techniques and the transmission curve, the error bar and loss for FX-1 310 are high due to the 21% loss of light seen at a working distance of 34 mm (see Figure 9b). On the ray traces, a constant loss was observed throughout all working distances and wavelengths. In optical simulations, the light traveling within a device or system does not vary unless other structures or objects affect the ray trajectory. Therefore, no change in the loss was observed with the working distance.

The data obtained in the presence and absence of the quartz tube ( Table 3) were compared to calculate the amount of light lost as it traveled through the walls of the quartz tube. In the case of FX-1 265, an average loss of 10 ± 0.55% was observed with a maximum of 11% loss (at a working distance of 14 mm) and a minimum of 8% (at a working distance of 19 mm). In the case of FX-1 310, where the highest amount of loss was observed (21%), this can be attributed to multiple reasons within the measuring system. In Figure 3b, it can be observed that there is a gap between the workbench (which moves the device to multiple working distances) and the measuring sensor ( on a motor gantry). It is possible that at higher working distances, the light is being lost owing to the gap and the surrounding environment within the working setup. In addition, in the case of these radiometric measurements on the half quartz tube, data were measured along the line of the tube rather than a rectangular space, which means that any light not within the length of the quartz tube, at the instant of time when the sensor captures the light signal, has not been measured. Table 4 summarizes the average loss observed with wavelength and the data from the comparison between the techniques.

**Table 4.** Data on loss of intensity in comparison with the transmission curve.

Wavelength / technique	Radiometry	Ray tracing	Transmission curve
**265 nm**	10 ± 0.55%	11%	11%
**275 nm**	9 ± 1.12%	10%	10%
**310 nm**	11 ± 2.84%	8%	8%

### Discrete ordinate method

To further understand the method and tool’s capability, this study conducted DOM to compare and validate the ray tracing-based modeling technique. As discussed earlier, the model was simulated only in an air medium for comparison and to understand the advantages and disadvantages of the models.

**Figure 10.**
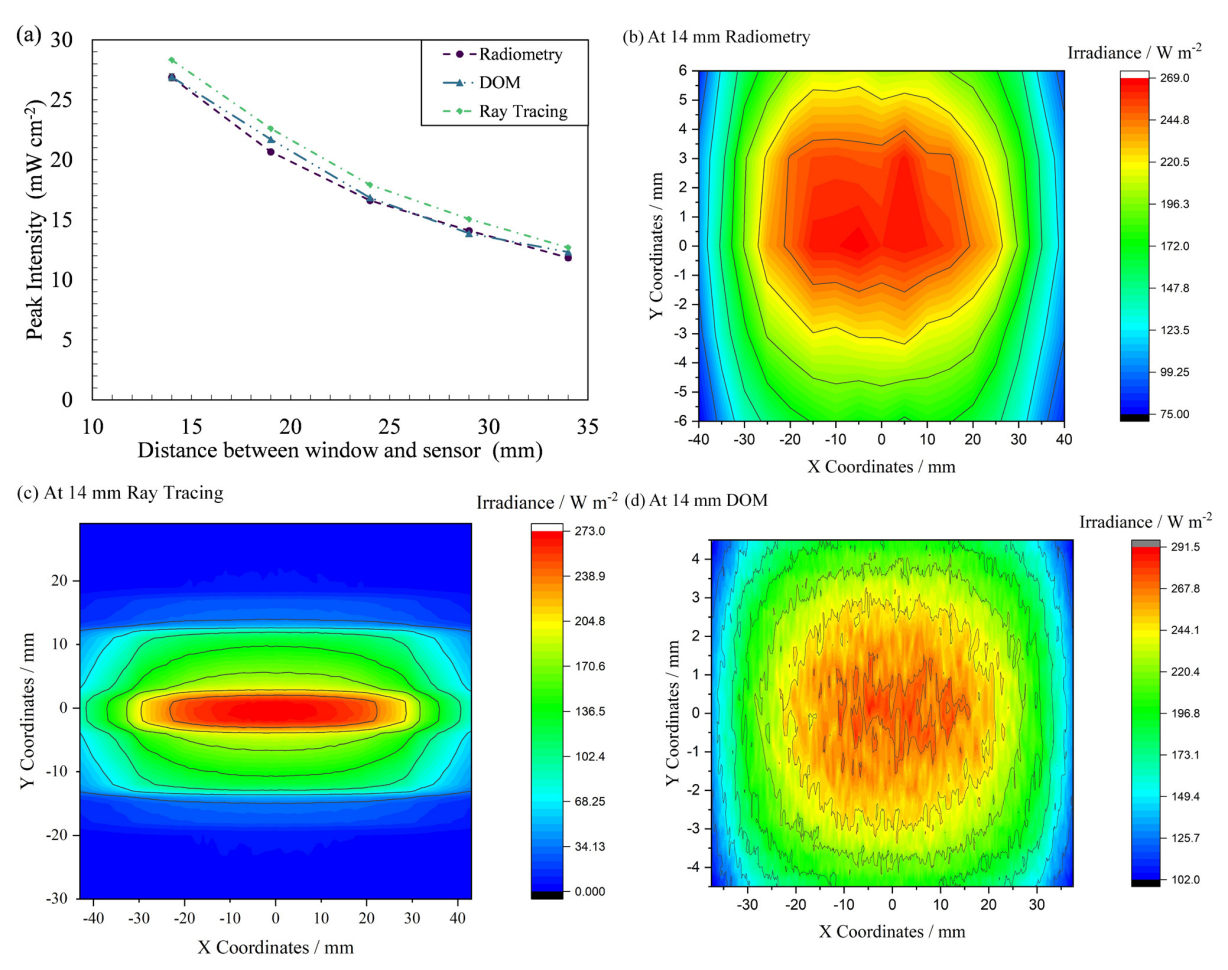
( **a**) Comparison between ray tracing, DOM and radiometry for FX-1 265, Uniformity plot obtained from ( **b**) radiometry, ( **c**) ray tracing interface and (d) DOM.

Table 5 summarizes the data obtained from simulations of FX-1 265. The data followed the same trend between the two simulations, and an average difference of 5 ± 0.86% was observed between the two simulation techniques. This difference could be due to the input value for direct irradiation of the DOM model. The input value for the DOM simulations was extrapolated from the radiometric measurements in Figure 8a. For optical ray tracing, the LED optical power was used as the input for the simulations. Data from DOM simulations can be seen to be closer to the radiometric measurements than that of optical ray tracing simulations (±1.71%). Figure 10a shows a comparison between the two simulation techniques, along with the radiometric measurements. The FX-1 275 and FX-1 310 results can be found in Additional Data (Figure S9 and Table S4 ^43, 49^) 

**Table 5.** Recorded peak intensity at multiple working distances between simulation tools.

Working distance (mm)	Recorded peak intensity (FX-1 265) (mW cm ^-2^)	
	Ray tracing	DOM
**14**	28.32	26.90
**19**	22.60	21.70
**24**	17.92	16.84
**29**	15.07	13.86
**34**	12.69	12.31

To further understand the difference between the two simulation techniques, uniform data were extracted. Figure 10b – d compare the uniformity plots obtained from the two simulations 14 mm away from the source along with the uniformity plot from radiometric measurements. Although all three techniques display a similar profile, owing to the size of the reactor design in DOM, the plot is cut off after the reactor size. In optical ray tracing, the analytical detector size does not impact simulations or simulation time; hence, a wider plot can be obtained. The uniformity plot shown in Figure 10d can be seen to be noisy, as the data obtained from DOM simulations is a collection of more than 10,000 data points, whereas the radiometry plot is a collection of ~600 high-resolution points measured by the radiometer at an instant of time during the measurements. The plot in Figure 10d can be smoothed by extracting data with fewer decimal points from the software. This has not been done to provide an overview and comparison between the solutions obtained from the respective simulation and experimental techniques.

Both the simulation techniques have their respective challenges and disadvantages. Both simulations are time consuming and need an elaborate amount of computational space for each simulation. In DOM, one of the main parameters controlling accuracy and precise simulations is the angular discretization of the light source. An increase in angular discretization increased the simulation time. On ray tracing tool, the main parameter dictating simulations is the number of rays within the simulation. For a rough understanding of the light emission and path within the system, a low ray count will work but for accurate simulations, higher ray count (greater than 10 ^6^) is recommended. The simulation time for ray tracing averaged up to 1 hour for high number of rays, whereas on DOM, for high angular discretization (greater than 15 X 15) of the light source, an average simulation time of 2–3 hours was observed.

The input of the number of rays is similar to conducting a mesh sensitivity analysis in DOM. In DOM simulations, it is important that the entire system be built as a solid in a specific space to obtain the results. In ray-tracing-based simulations, the design of the system is based on an X-Y-Z coordinate system that enables easier design and can be built according to the design and dimensions. In both simulations, the main body was simplified to reduce simulation time and processing. As in the case of this study, the entire body of the device was not designed, and only the light head section, as this is the only part that contributes to simulating light travel. Both methods can simulate the presence of water and air in front of the source; however, it was observed that the ray-tracing method is easier than DOM simulation for both the design and simulation steps. Unlike DOM, each part can be designed independently; hence, any issues incurred during or after simulations can be detected as specific to the part and not the system as a whole. This helped reduce the time for correction and changes, whereas for DOM, the time required to understand the issue and make changes was significantly higher. In simulations where light source data are important, an advantage of ray tracing over DOM simulations is the useful input of the radiation pattern of the light source. In ray tracing, the light source can be programmed according to the manufacturer’s datasheet and optimized for the device design. The ray tracing technique is mainly an optical tool; hence, particle tracking simulations cannot be conducted in this tool, but can be done on DOM ^36^.

### Characterization using actinometry

Once the ray-tracing model was validated in an air medium, it was developed to understand its behavior in a water medium. To achieve this, multiple changes were made within the designed model, as discussed earlier.

**Figure 11.**
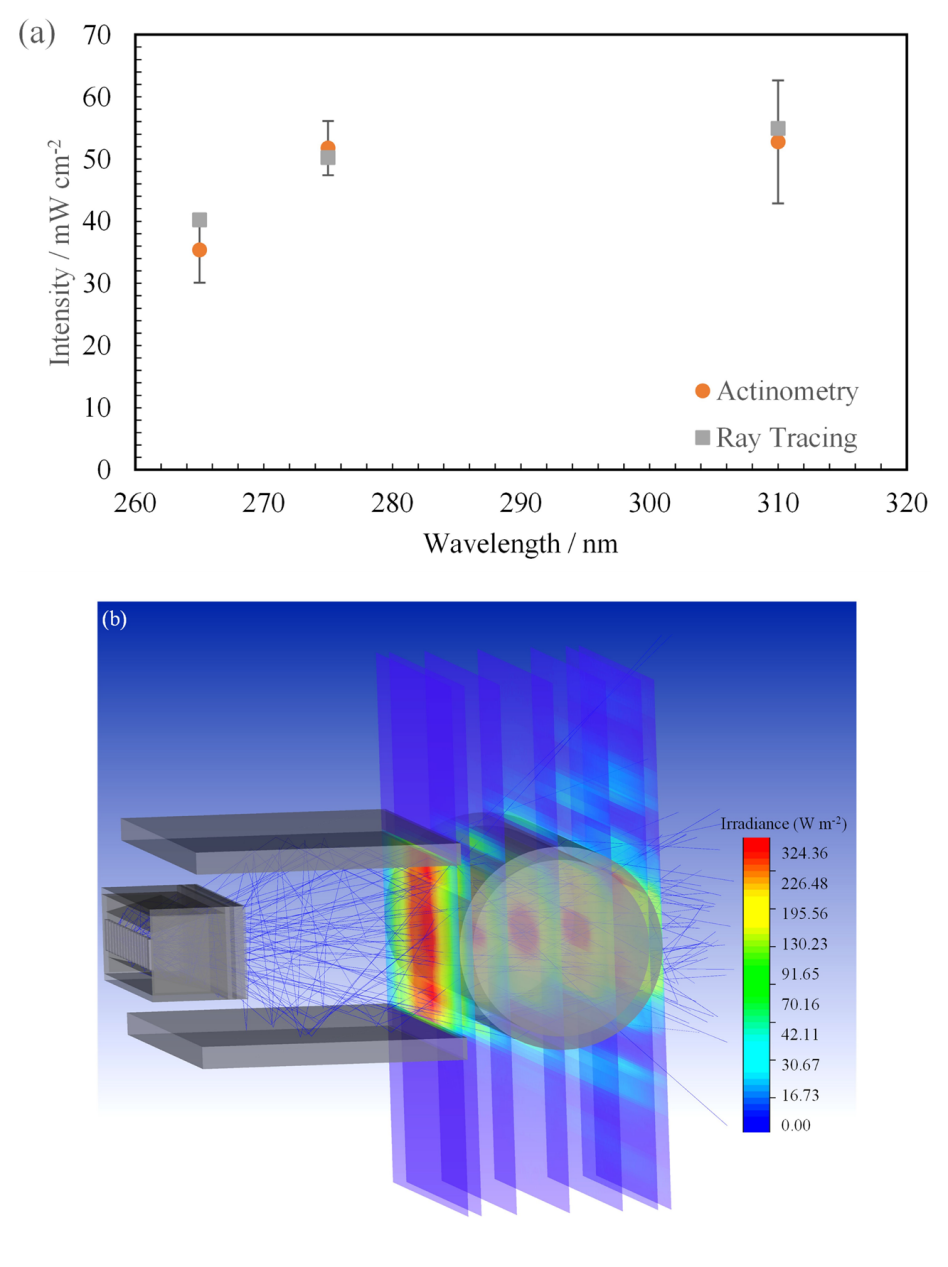
( **a**) Plot of intensity with wavelength between the two techniques, ( **b**) Isometric view of changed ray tracing model.

- To design a tube on the tool, multiple “object types” were considered and tried. A hollow cylinder-type object was not readily available; hence, to build the quartz tube within the model, the Boolean operation technique was applied. Two cylinders were designed using Boolean operations, and the inner cylindrical area was subtracted from the outer solid cylinder to obtain a hollow cylinder with the dimensions of the quartz tube within the UV fixture. In doing so, the software enabled the input of two materials for each cylinder. The inner cylinder material was changed to “water” while the outer cylinder material was set to “quartz” ^44^.
- During actinometry, the device was placed within the UV fixture, and samples were taken. Within the UV fixture, the environment was different from the standard workbench setup shown in Figure 3a. Multiple design bodies and/or surfaces exist, ensuring that little light is lost within the system. To ensure that the simulation model replicated the actinometry measurements, two extra design bodies on the top and bottom of the light source body (see Figure 11b) were designed according to the UV fixture model.
- Analytical detectors or measuring points in the model have only been placed within the tube, and the software assumes that the remaining simulation space is air.
- The refractive index of water within the simulation was changed for each wavelength based on data from the literature ^45^ and is listed in Table 2.

Actinometric measurements were conducted on 3 separate days to ensure that reproducible and repeatable data were obtained. The quantum yield used to convert the measured data to mW cm ^-2^ has been listed in Table 2. The technique was applied to all three wavelengths in this study and was conducted only at a working distance of 14 mm from the center of the quartz tube. This technique was used to validate the ray-tracing model and check whether the simulation agreed with the measurements. Conducting actinometry for each working distance can be time-consuming, whereas the simulation displays data for all points of interest in less time. The measured data from the actinometric measurements are shown in Table 6 in comparison with the extracted peak intensity data at the center of the quartz tube in the simulated model. It can be seen that both the measured and simulated data are in close agreement with each other i.e., within the error range of the actinometric measurements for all sources studied (see Figure 11a). The FX-1 275 can be seen to have the closest agreement 51.76 ± 4.37 mW cm ^-2^ in actinometry to 50.22 mW cm ^-2^ on the ray tracing model. The difference between the two techniques was approximately 3%. Data on actinometry measurements can be found in Additional Data (Table S5 and Figure S10 ^43, 49^).

**Table 6.** Data on comparison between actinometry measurements and ray tracing.

Device	Measured intensity (mW cm ^-2^)	
	Actinometry	Ray tracing
**FX-1 265**	34.97 ± 5.51	40.22
**FX-1 275**	51.76 ± 4.37	50.22
**FX-1 310**	52.77 ± 9.88	54.93

Upon observing considerable agreement between the two techniques, the effect of water has been quantified using the same model seen in Figure 11b. Simulations have been conducted at multiple working distances within the UV fixture in air and water mediums. Table 7 summarizes the comparison between air medium and water medium for FX-1 265. It can be seen that there is an increase in irradiation at all working distances when light passes through a water medium. It is known that very little light in the ultraviolet range is absorbed by water and hence the increase is predominantly due to multiple optical phenomena occurring within the medium as the light propagates ^24^. As the light passes through the water medium, it undergoes refraction and reflection, meaning multiple rays can be interacting at a given point within the simulation and system. Also due to the design ( Figure 2a) and material (Aluminum) of the UV fixture, very little light is lost and there is a high probability that any light not entering the quartz tube directly is reflected back. Within the set-up, due to supporting structures, light can be seen to reflect off the surface of the fixture and measured within the simulation and in actinometry as seen in the ray traces in Figure 11b.

**Table 7.** Comparison between measured intensity in air and water medium for FX-1 265.

Working distance (mm)	Measured peak intensity. within the set-up (mW cm ^-2^) (Ray tracing)		% Increase in irradiation
	Air medium	Water medium	
**14**	25.28	40.22	37.15%
**19**	20.18	37.42	46.07%
**24**	16.00	33.91	52.82%
**29**	13.45	37.76	64.38%
**34**	11.33	31.32	63.83%

**Figure 12.**
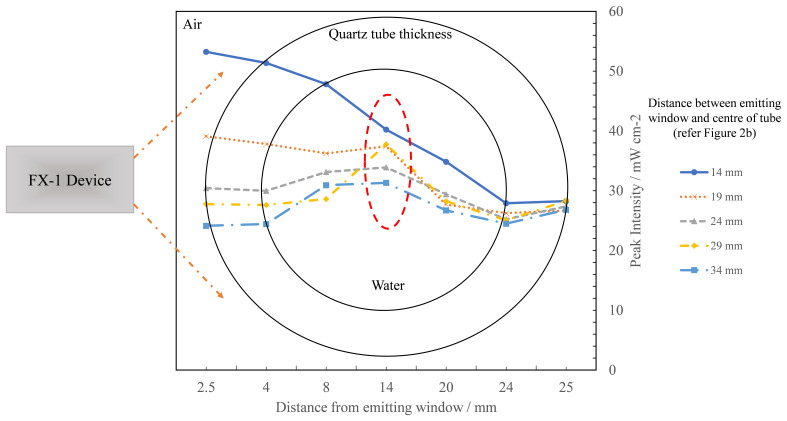
Plot of change in peak intensity as light propagates through the quartz tube within the UV fixture at multiple working distances for FX-1 265.

By creating multiple analytical detectors along the diameter of the quartz tube, the model also enabled an understanding of how the light intensity progresses from the window until the end of the quartz tube (see Figure 11b). Figure 12 traces the measured peak light intensities as the light passed through the system designed at multiple working distances from the emitting window of the device. At 14 mm, the peak intensity profile with working distance follows nearly the same trend as expected (a drop is expected in the maximum peak intensity with distance) but at other working distances the trend observed is significantly different. It can also be seen that once the light enters the quartz tube, the highest intensity simulated is seen in the center of the quartz tube (red circle in Figure 12). This is due to multiple reasons, including reflections from inside the UV fixture body as the distance increases between the source and tube, total internal reflections in water, light reflecting from opposite walls of the fixture and due to the optical phenomenon occurring as the light passes through the thickness of the quartz tube (3 mm).

### Analysis of a 4-wavelength germicidal system

**Figure 13.**
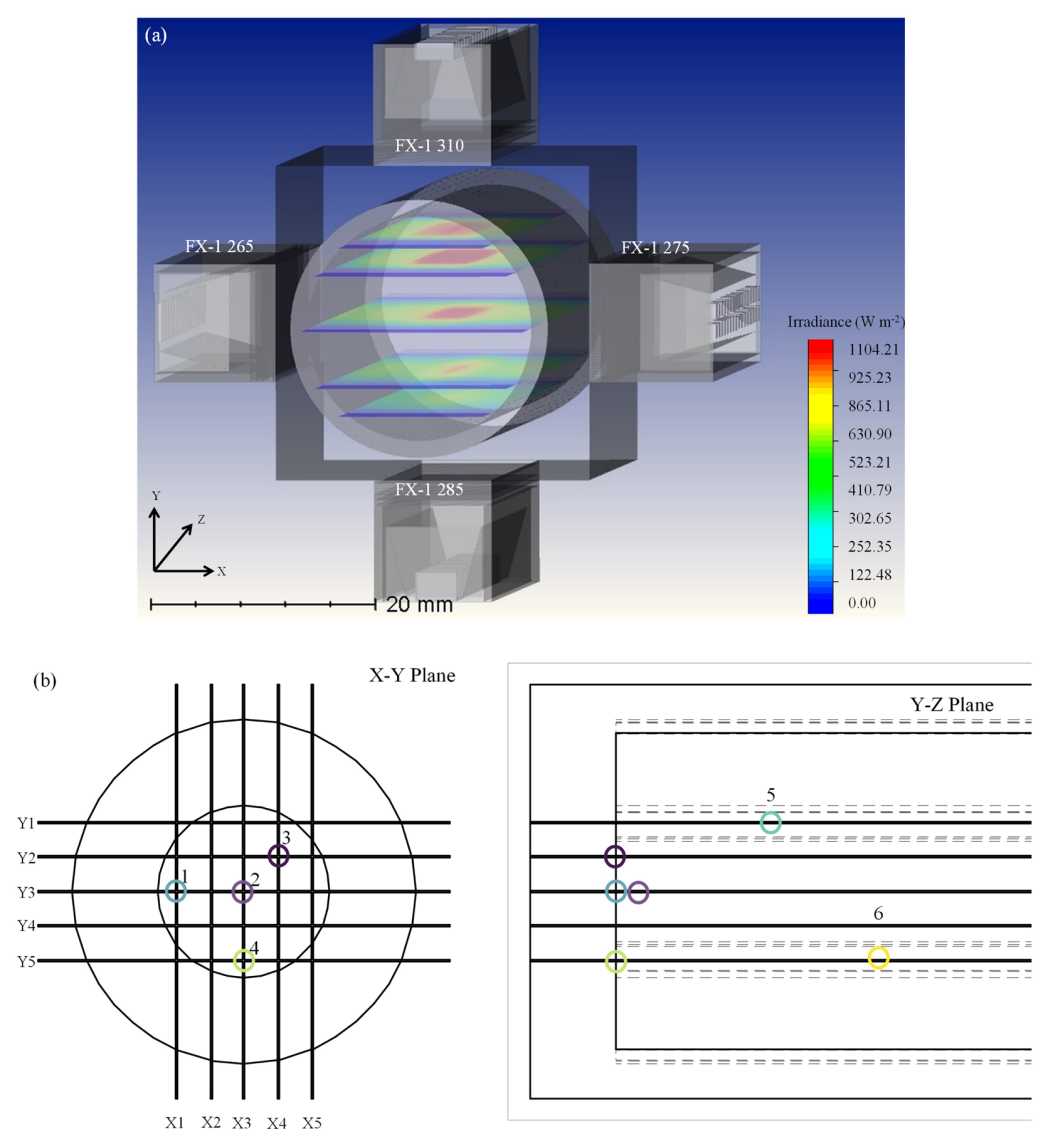
( **a**) Ray tracing model for a complex system involving 4 FX-1’s operating at different wavelengths and ( **b**) Depiction of points of interest in a complex system.

From the previous sections, it is clear that the ray-tracing tool estimates intensity values within the error ranges of the validated and known methods in the literature. In the case of a complex system involving more than one wavelength, the experimental methods do not provide any information or data on the contribution of individual wavelengths to the overall intensity measured by the respective method. It was observed that ray-tracing provided these data. To design a complex system involving multiple wavelengths and devices, the tool allows the use of mirror and rotation functions to reduce the modeling time in this study. To simplify the amount of time required for simulations and to better understand the observed data, two simulations were conducted. The first simulation is shown in Figure 13a, where the analytical detectors were parallel to the *x-axis* to obtain data from FX-1 310 and FX-1 285. For the second simulation, the analytical detectors were moved parallel to *z-axis* to obtain the data from FX-1 265 and FX-1 275. The data from the two simulations were combined to provide information on the light propagation within the tube. For example, consider points 1-6 in Figure 13b, Table 8 summarizes the data observed in these simulations, and the simulation time for such a complex model was seen to be about 3-4 hours. This simulation provided insights into the amount of light and wavelength reaching the points of interest within the tube.

**Table 8.** Simulated intensity in water at different points within the tube.

Point number ( Figure 13b)	Total intensity (mW cm ^-2^)	Spectral intensity in mW cm ^-2^ (% )			
		FX-1 265	FX-1 310	FX-1 275	FX-1 285
**1**	135.38	50.63 (37%)	20.62 (15%)	13.78 (10%)	50.35 (37%)
**2**	199.60	40.45 (20%)	54.66 (27%)	50.22 (25%)	54.27 (27%)
**3**	147.64	37.25 (25%)	35.22 (24%)	29.80 (20%)	45.36 (31%)
**4**	160.03	46.55 (29%)	17.43 (11%)	34.65 (22%)	61.40 (38%)
**5**	131.11	16.35 (12%)	44.26 (34%)	27.55 (21%)	42.95 (33%)
**6**	111.92	22.68 (20%)	10.45 (9%)	27.37 (24%)	51.41 (46%)

Ray tracing has been shown to be effective and efficient for understanding the path of light as it travels through the system. The optimization of a system needs to be dealt with on a case-by-case basis and specific to the design of the system. For germicidal systems, it has been reported that a range of 250-270 nm is optimal for effective disinfection of water ^54^. For example, in this study, using data from primary models in air and water, simulated data provided inputs on the behavior of each wavelength within the designed system. When irradiated simultaneously, each wavelength contributed a certain percentage of irradiation to the overall intensity. As shown in Table 8, at point 2 (center of the quartz tube), FX-1 285 irradiates the majority of the total irradiance, followed by the FX-1 310 device. To optimize the system and achieve effective and efficient disinfection of water, FX-1 285 and FX-1 310 can be moved within the UV fixture to 24 mm away from the center of the quartz tube, thereby reducing the contribution of the respective wavelengths to the total irradiation (24.82% and 20.06%, respectively). The effectiveness of a germicidal system can be enhanced depending on the type of microorganism being evaluated and disinfected.

It is worth noting that the above study was conducted for a static model system; however, there are other factors, such as the flow state and water matrix condition, that can directly influence the light attenuation process that need to be considered for further studies. At the same time, the effect of quartz material needs to be studied in further detail by evaluating factors such as the effect of tube thickness, material properties, and design.

## Conclusions

In summary, the present study reports for the first time the application of an optical ray tracing method to predict irradiation, reaching the point of interest as light propagates through water in a germicidal system. The optical ray-tracing modeling method was compared with radiometry, DOM, and ferrioxalate actinometry. The results in comparison to these techniques have been seen to be in close agreement (±6%). This proved that the proposed method can be used to overcome the major challenges faced during the measurement and simulation of irradiation in water. The study also quantified the effect of quartz material on irradiation in the UV range of the light spectrum and observed a decrease in light intensity by 10 ± 0.55% for FX-1 265. The comparison between the light transmission curves for the fused quartz material used in this study provided an understanding of the light behavior at multiple working distances as it passed through the 3 mm thickness of the tube. Constant light loss was observed in the simulations, whereas variable light loss was observed in the radiometric measurements. The study also validated measurements in water using ferrioxalate actinometry and provided an understanding of the increase in light intensity in a water medium due to total internal reflections and scattering of light in water. This study found an average 52% increase in light intensity across multiple working distances in water for FX-1 265 with respect to the air medium in the presence of a quartz tube. The method was further used in a 4-wavelength complex system and enabled a better understanding of system design. The obtained data showed individual wavelength contributions at multiple points of interest within a complex system. Although the predictions of radiant intensities by optical ray tracing simulations are higher than those of the experimental and other simulation methods, the differences are within the error range. Each application must be worked on a case-by-case basis. In conclusion, this method provides a valuable understanding of how the light source propagates through the system, how to optimize the light irradiation within the designed system, and the difference between air- and water-based systems.

## Ethics and consent

Ethical approval and consent were not required for this study.

## Data Availability

Zenodo: Dataset of paper "Comparison of radiant intensity in aqueous media using experimental and numerical simulation techniques" https://doi.org/10.5281/zenodo.10054947 ^49^ This project contains the following underlying data: Relative spectral intensity for each light source as recorded by ILT RAA4 spectroradiometer. Material and optical properties of the quartz tube. Wavelength specific refractive index and quantum yield in water. Comparison of ray tracing data vs experimental data (FX-1 265). Peak intensity vs working distance (in air). Comparison between radiometry and ray tracing in the presence of a quartz tube for FX-1 265. Recorded peak intensity at multiple working distances using radiometry and ray tracing. Comparison between average light loss at multiple distances in radiometry, ray tracing and transmission curve due to quartz material. Percentage light loss for all wavelengths at multiple working distances due to quartz material. Loss of intensity and transmission curves. Recorded peak intensity at multiple working distances between simulation tools. Comparison between ray tracing, DOM and radiometry for FX-1 265, Uniformity plot obtained from radiometry, ray tracing interface and DOM. Intensity vs wavelength for actinometry measurements and ray tracing. Comparison between measured intensity in air and water medium for FX-1 265. Change in peak intensity as light propagates through the quartz tube within the UV fixture at multiple working distances for FX-1 265. Simulated intensity in water at different points within the tube. Data are available under the terms of the Creative Commons Attribution 4.0 International license (CC-BY 4.0). Zenodo: Supplementary material of paper "Comparison of radiant intensity in aqueous media using experimental and numerical simulation techniques" https://doi.org/10.5281/zenodo.10055089 ^43^. This project contains the following extended data: Information on the design layout for wavelengths 265 nm, 275 nm, and 310 nm. Mesh sensitivity analysis results and optimum mesh data for simulations. Rollover tests. Optical model comparisons. Effect of a quartz tube on peak intensity. Radiometry measurements for various working distances and wavelengths. Simulation results for various working distances and wavelengths. Ferrioxalate actinometry data for various working distances and wavelengths. Data are available under the terms of the Creative Commons Attribution 4.0 International license (CC-BY 4.0).
