# Comparison of radiant intensity in aqueous media using experimental and numerical simulation techniques

**DOI:** 10.12688/openreseurope.16812.2

**Published:** 2024-10-18

**Authors:** Adithya Pai Uppinakudru, Cintia Casado, Ken Reynolds, Simon Stanley, Cristina Pablos, Javier Marugán

**Affiliations:** 1 Universidad Rey Juan Carlos, Móstoles, Community of Madrid, Spain; 2 Prophotonix, Cork, Ireland

**Keywords:** actinometry, discrete ordinate, optical design, radiometry, ray tracing, ultraviolet light

## Abstract

Accurately modelling the propagation of radiant intensity in aqueous environments poses significant challenges for both academia and industry, due to complex interactions like absorption, scattering, and reflection. This study aims to improve the accuracy of optical modeling in water-based systems by comparing experimental data with numerical simulation techniques, addressing the need for more reliable simulation methods in multiple applications like treatment of water and environmental monitoring.Implementation has been done by analyzing how the method compares with the discrete ordinate method, radiometry, and actinometry. The study further quantifies the effect of the photoreactor quartz tube on measured intensity for multiple wavelengths. Losses in light intensity are estimated to be 10 ± 0.5% for FX-1 265 source. In contrast, the simulation in a water medium showed an increase of up to 64% in the light intensity delivered to the central part of the tube due to internal reflections and scattering. Model predictions from ray tracing successfully compared with the Discrete Ordinate Method (DOM) and experimental data (within ± 6%), ensuring the accurate design of complex systems for water disinfection. The data from simulations is seen to tackle challenges faced in complex radiation modeling and demonstrates that the method can be utilized as a useful tool for optimization and prediction.

**Table:** 

Nomenclature			
*Abbreviations*		*Symbols*	
**DOM**	Discrete Ordinate Method	**A (λ), *abs***	Decadic absorbance (dimensionless)
**IUPAC**	International Union of Pure and Applied Chemistry	**q ^0^_p_**	incident photon flux
**LED**	Light Emitting Diodes	***k***	Ray direction cosine vector
**PCB**	Printed Circuit Board	***l, m, n***	Direction cosines of unit vector $\vec{k}$
**RTE**	Radiative Transfer Equation	**N**	Unit normal vector of the surface at the point of contact
**UV**	Ultraviolet light in the wavelength range of 100–400 nm	**T**	Temperature (K)
		***x, y, z***	Coordinates of ray (m)
*Greek Letters*		*Vector Symbols*	
**λ**	Wavelength (nm)	${\text{I}}_{\lambda ,\vec{\Omega }}$	Intensity of photons with wavelength λ and direction $\vec{\Omega }$
**ρ**	Density (kg m ^-3^)	$\overset{⌢}{\text{k}}$	Direction of the ray
**σ**	Stefan Boltzmann constant (W m ^-2^ K ^-4^)	$\vec{\Omega }$	Unit vector in the direction of radiation propagation
**σ _k_**	Volumetric scattering coefficient (m ^-1^)	$\vec{\text{r}}$	Directional position of the ray in a cartesian coordinate system
**Φ**	Wavelength averaged primary quantum yield (mol Einstein ^-1^)		
**Φ(λ)**	Number of molecules changed, formed or destroyed divided by number of absorbed photons		
***Ω***	Solid angle of radiation propagation about the direction $\vec{\Omega }$		

## Introduction

The recent increase in demand and interest in UV light devices for disinfection has led to increased research to optimize the process and achieve better disinfection rates. One key aspect of evaluating and optimizing a UV reactor's efficiency is understanding the path of light as it travels through the system. Many methods have been applied theoretically and experimentally to understand the path of ultraviolet light as it travels through a specific medium since the idea of its germicidal effectiveness was observed by Downes *et al*. in 1877 ^1^. Experimental techniques like radiometry and actinometry are physical techniques that require the use of a physical validation set-up for measurements of light. Radiometry is widely used in the lighting industry for measurement of light sources ^2^. This technique employs a radiometer (consisting of a sensor or detector and a signal processing unit), which when subjected to light, measures the amount of light reaching its receiving surface. While very useful in air mediums, it faces challenges in water-based environments. Very few detectors and radiometers exist in the market that are waterproof or can measure light in water ^3– 5^. These detectors are of high cost and/or require special attachments, which could cause other issues, such as leaks, inaccurate measurements etc., within the system. Another experimental tool employed in the laboratory is chemical actinometry. This technique involves the use of chemicals that absorb photons as the light passes through the system leading to a measurable chemical reaction from which the number of photons absorbed is estimated using a known quantum yield ^6, 7^. Actinometry, while useful to measure light, does not provide inputs to help optimize the light source as the measured data only signifies the cumulative number of photons absorbed by the used chemicals as it is exposed to light over a period of time.

As discussed above, experimental techniques have worked well in an air medium, however in a water based medium, multiple operational challenges have been faced by operators ^8^. To overcome these challenges, simulation techniques have been employed to understand the radiant intensity in a water medium. Simulation techniques like discrete ordinate are models in a virtual environment that help understand theoretical light irradiations acting at the point of interest.

Over the years, researchers have considered multiple simulation approaches. Two main approaches have been used in the literature – Eulerian (volumetric reaction rate based) and Eulerian-Lagrangian (particle tracking based) frameworks ^9– 12^. Several models have been proposed for radiation distribution and to evaluate the kinetic rate constants of microbial inactivation ^13– 16^. It has been shown that reactor dynamics, radiation, and kinetics can be solved using simulation packages such as COMSOL, ANSYS or OpenFOAM based on Computational Fluid Dynamics (CFD) for reactor modeling. Within the Eulerian framework, the conservation equations of mass and momentum are solved. The comparison of the average particle (in this case, simulated microorganisms) values helped determine the overall performance of the UV reactor ^17^. Within the Lagrangian simulation framework, the trajectories of microorganisms (considered as dispersed particles) are computed considering the Newtonian equation of motion. The inactivation is determined using the accumulated light energy within the described path ^17^. Unluturk *et al*. 2004 ^18^ and Wright *et al*. ^10^ used the combined Eulerian-Lagrangian framework to simulate UV photoreactors for microbial water disinfection.

Keshavarzfathy *et al*. ^19^ elaborated on the need to conduct studies on design concepts that lead to a better understanding of the hydrodynamic interactions and reactor performance. The research studied the development of a model for simulation of UV LED based reactors in the Eulerian framework. The Monte Carlo method is another approach considered in literature ^20, 21^. This approach is a stochastic method that allows for a flexible geometry and adapts well to complex statistical simulations ^22^. The technique involves tracking the trajectory of a large number of photons and computing the location where they are absorbed in a 3-dimensional space. Busciglio *et al*. ^23^ further considered a probabilistic approach to radiant field modeling in a system and validated the model using Monte Carlo simulations. The research found significant agreement between the two techniques. The above discussed approaches can cover all domains within the reactor if enough particles are taken into account for analysis. Nevertheless, the subject of radiation modeling and transfer in the type of media has been approached from multiple directions in literature and multiple challenges have been observed. Shah *et al*. reviewed two methodologies for modeling – SURF (simultaneous UV fluence rate and fluid dynamics) and TURF (three step UV fluence rate and fluid dynamics) and concluded that the CFD models can predict the dosage received by water better than applying the average dosage to the system based on the power of lamps ^24^. The performance of a reactor depends on multiple factors within the system, including the interaction between radiation type, radiation dynamics, and the design of reactor ^25^. In a 3-dimensional domain, obtaining an accurate prediction of radiant field and intensity reaching a point of interest requires powerful computing capability and space.

Although the above discussed simulation and experimental techniques are used for their respective applications, these models and tools also lack some inputs that are necessary for accurate and valid prediction of light irradiation in a flow based system due to the inability to incorporate certain parameters that play a key role in the amount of irradiation reaching the point of interest, including: i) Accurate source modeling to accommodate the radiation pattern of the light source; ii) Scattering and reflections incurred due to design of the emission system and turbulent flow of water through the system and iii) Inputs on how to optimize the light reaching the point of interest given that UV LEDs operate at low efficiencies. However, the exact knowledge of optimizing the amount of light reaching the point of interest remains unknown in most cases and these parameters have been continuously evolving with time. Light sources have moved from mercury lamps to use light emitting diodes, which are much smaller than the former, making the recent germicidal systems less bulky. In this present study multiple methods have been utilized to validate the modeling predictions.

In this study, a method for prediction of radiant intensity reaching a point of interest within a water-based medium is studied using optical ray tracing by considering the actual radiation profile of the UV LED selected, optical phenomenon occurring within the medium, design optimization, and change in intensity at interfaces to overcome challenges faced in both experimental and simulation techniques currently used. Ray tracing technique is mainly used in pre-production stages of light source manufacturing. It has been widely debated as to how effective ray tracing is in comparison with the traditionally used Monte Carlo algorithms. Li *et al*. ^26^, in their work on comparison between the two techniques for spine lesions, observed that ray tracing significantly overestimates the volume of target covered by the dose for one case but saw that the estimated dose difference was within 3% between the two techniques for the three other cases studied. The main difference in the former case was the presence of multiple air cavities in the study. In conclusion, the authors mention that the ray tracing technique is “adequate” for use in most cases, but it would need to be validated with other techniques for further use of simulated data ^26^. In a similar type of study comparing ray tracing with reverse Monte Carlo method for application to a GEO orbit, Benacquista *et al*. concluded that the ray tracing method is fast but presents intrinsic limitations that need to be verified before further use ^27^. Monte Carlo simulations have been used in literature for reactor modeling and have been proved to be time consuming while also requiring a large computational space ^28– 30^.

To the author’s knowledge, this study is the first to employ use of ray tracing in a germicidal system and attempts to trace the path of ultraviolet light as it propagates through the system and water medium. There exist multiple tools from different manufacturers that employ ray tracing theory like 3Delight ^31^, POV-Ray ^32^ and ZeMax ^33^. ZeMax optic studio has been used for design and simulation of the system for this study. Given the limitations seen in the literature, this study analyses the validity of the method via comparison with existing techniques employed in academia and industry. The study conducts a step wise analysis of the designed system by comparison with radiometry in an air medium and uses obtained data to quantify the effect of a quartz tube on the irradiation in air. This quantification provides insights into the amount of light lost at multiple working distances within this method. DOM simulations commonly employed in photoreactors simulations ^34– 37^ have be used to understand the steps and challenges between the two simulation techniques. The study also develops a model to simulate the presence of water and compares it with a lab-based method used to calculate the number of photons entering water i.e., ferrioxalate actinometry and compares the increase in radiant intensity as the light passes through the set-up. Finally, a model has been built to provide insight into the radiant energy distribution within a complex system of 4 wavelengths and an understanding of how light intensity changes as it propagates within the tube.

## Materials and methods

### LEDs source, fixture design and spectral characterization

**Figure 1.**
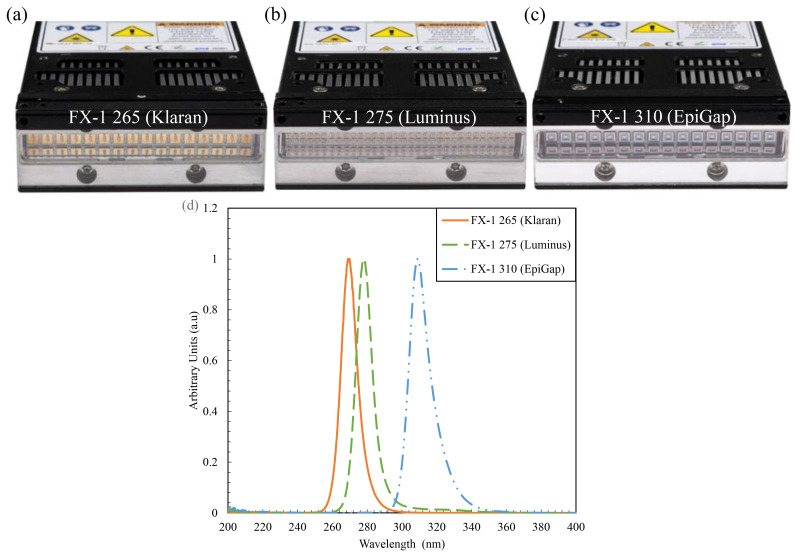
( **a–c**) FX-1 sources and ( **d**) Relative spectral intensity for each light source as recorded by ILT RAA4 spectroradiometer.

Three LED light sources of spectral emission in the UV-B and UV-C ranges have been selected for this study. The LEDs chosen for this work were 265 nm (KL265-50U-SM-WD, 70mW, Klaran), 275 nm (XBT-1313-UV-A150-AG270-00, 8mW, Luminus) and 310 nm (EOLS-310-697, 50mW, EpiGap) as a part of the REWATERGY project (Project N. 812574). To conduct analysis and experiments, the LEDs were built onto the COBRA Clean FX-1 (here onwards called FX-1) supplied by ProPhotonix (See Figure 1 a–c). Spectral measurements have been made to ensure that the emission of the LEDs is within the scope and interest of this study using a spectroradiometer (2003357U1, International Light Technologies) with the RAA4 coupling optic. Figure 1d shows the spectrum of each LED relative to their respective peak wavelength.

The FX-1 series by ProPhotonix has been used as the device to test the LEDs selected for the purpose of this study ^38^. The device (emitting window size of 76.8 mm X 28 mm) has been designed to fit the UV LEDs chosen according to their respective footprint. The FX-1 265 and FX-1 310 accommodated 16 LEDs each ( Figure 1a, c), while due to the size of the 275 nm LEDs (1.35mm *1.35 mm), 64 LEDs were accommodated on FX-1 275 ( Figure 1b). Light is emitted and controlled by a controlled conditioner driver that uses 48V DC safe current. The driver has a micro-controller on the printed circuit board (PCB) which monitors the temperature of the substrate and applies cooling via the fans on the device. The driver helps maintain a safe electric current, to not short or burn out the LEDs. It uses a 0–10 volts analogue signal that corresponds to 0–100 percent intensity range.

**Figure 2.**
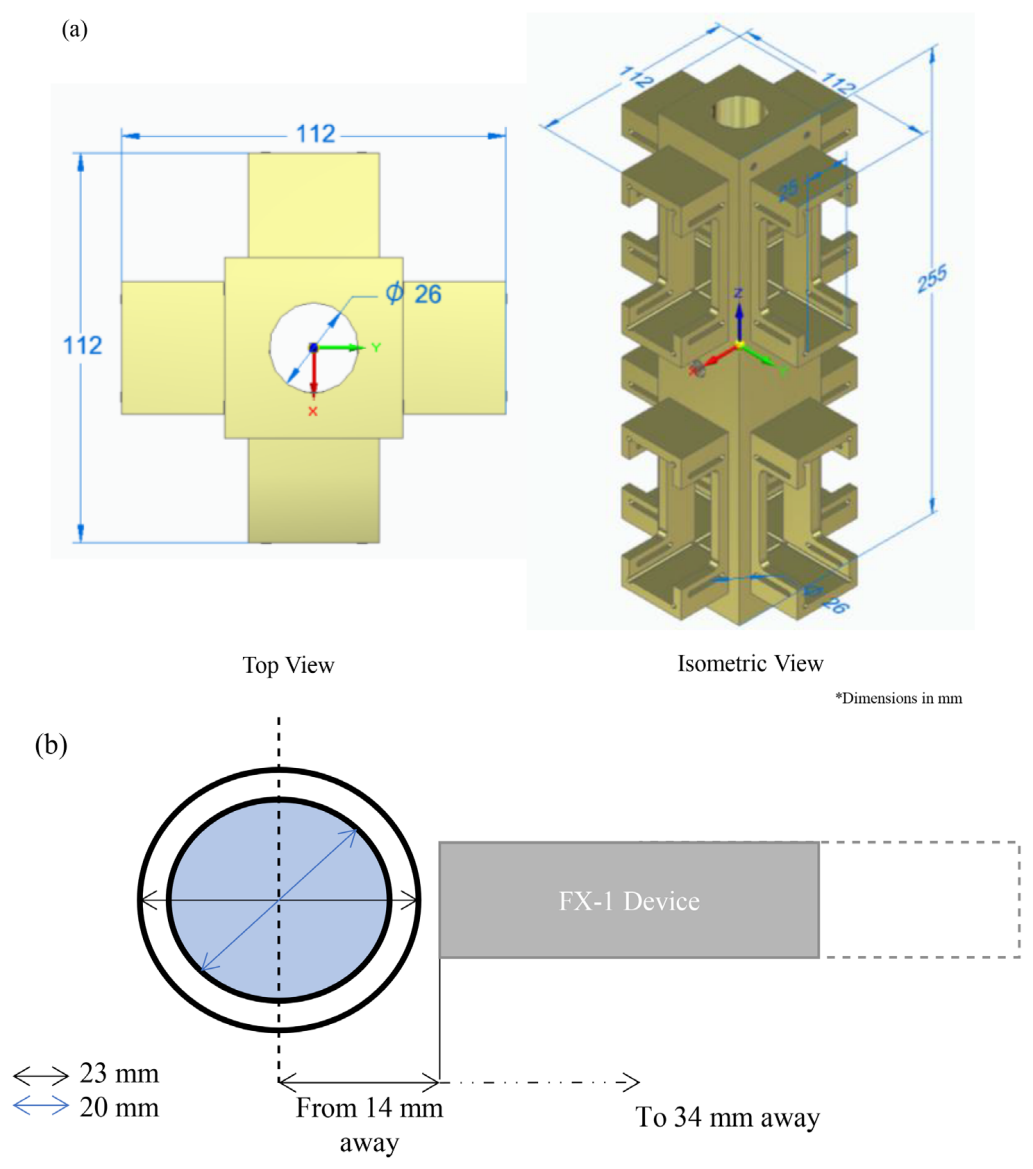
( **a**) UV Fixture and ( **b**) Representation of distance from the center of the tube to the source window.

To conduct actinometry and further tests, a custom designed UV fixture was manufactured (See Figure 2a). The UV fixture can accommodate up to 8 FX-1’s and a quartz tube. The quartz tube can be connected to the sampling tank and outlet to enable a flow through system (see Figure 5a). The quartz tube used for this study has an external diameter of 23 mm, an inner diameter of 20 mm and a length of 100 mm (FAB028553, Multi-Lab Ltd). The devices, when mounted on the UV fixture, can be moved to multiple working distances (from 14 mm to 34 mm away from the center of the quartz tube – see Figure 2b representation). The UV fixture, seen in Figure 2a, is made up of aluminum material to ensure any light lost can be reflected back into the quartz tube.

**Figure 3.**
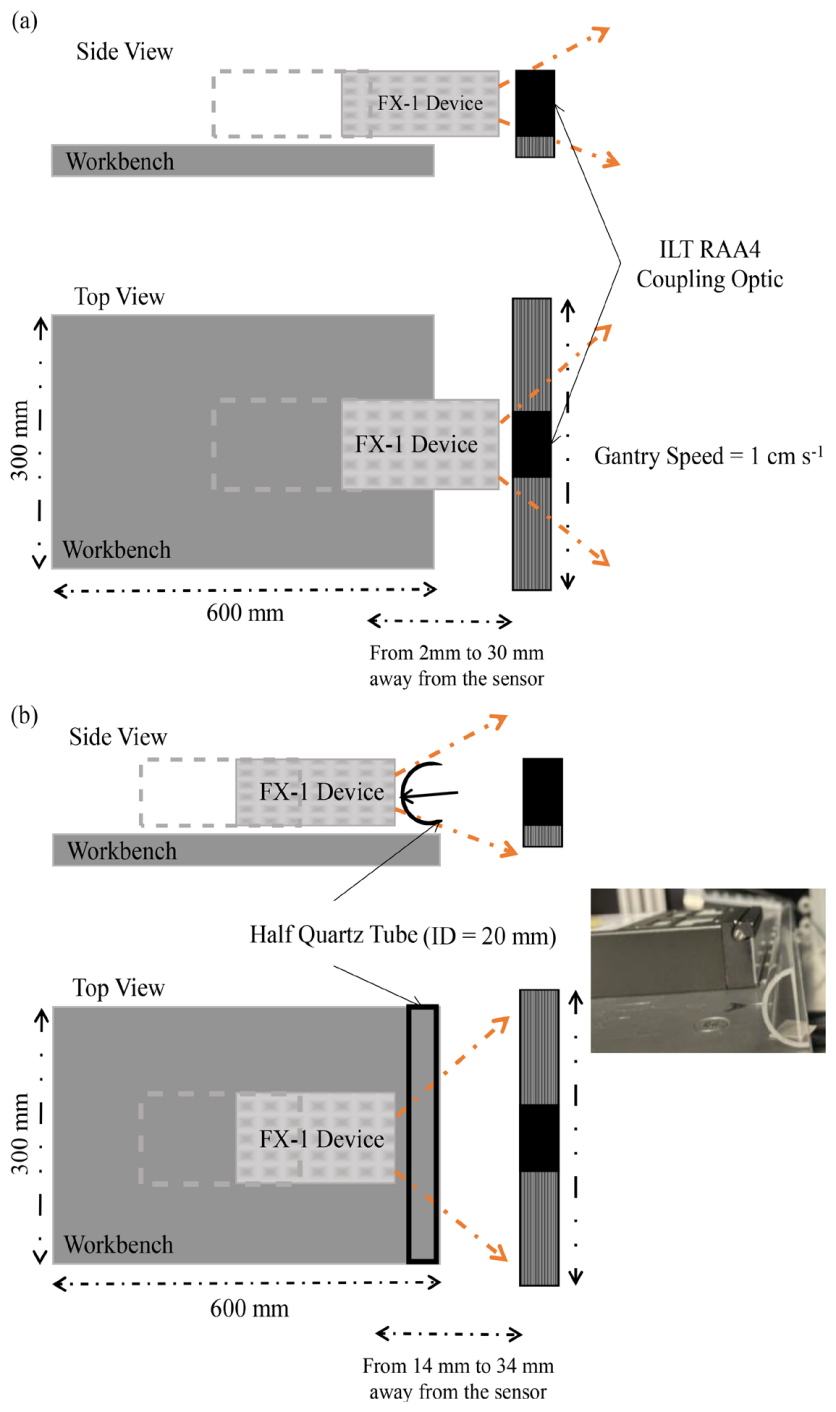
( **a**) Representation of X-Y tester for radiometry, ( **b**) Representation of X-Y tester for half quartz tube radiometry (Not to scale).

To characterize and quantify the intensity of light emitted from the device, a spectroradiometer has been used. The spectroradiometer is the ILT 950UV series measuring radiation in the range of 210 nm to 1100 nm ^39, 40^. To understand the complete emission profile of the device and other characteristics, an X-Y based motor gantry tester was used ( Figure 3a). The tester consists of a workbench (where the device can be mounted and moved to multiple working distances away from the optical sensor) and a motor gantry which supports the optical sensor and moves as required in longitudinal and lateral directions. The entire set-up is controlled using a LabVIEW VI that has been set-up and programmed to collect data from measurements for further analysis. The entire set-up is enclosed in a black box to avoid exposure to harmful UV radiation.

### Set-up to quantify the effect of quartz tube

One of the objectives of this study was to quantify the effect of quartz tube and compare it with the results obtained from the ray tracing model. To do this, a custom manufactured half-quartz tube cut along its length has been used. The tube is from MultiLab Ltd (FAB027469, 23 mm outer diameter, 20 mm inner diameter and 100 mm length). Figure 3b is a schematic representation of the change in set-up for this experiment and Table 1 summarizes the quartz tube properties, including the transmittance data as obtained from the manufacturer. The quartz tube and device have been placed as per dimensions on the UV fixture discussed above ( Figure 2a) and tested at the same working distances as available on the fixture.

**Table 1.** Material and optical properties of the quartz tube.

Property	Typical Value
Density (ρ)	2.2 × 10 ^3^ kg m ^-3^
Refractive Index	1.485
Transmittance	At 265 nm - ≈0.89 At 275 nm - ≈0.90 At 310 nm - ≈0.92

### Optical modeling of the devices with ray tracing

ZeMax Optic Studio has been used for the optical simulation. This tool is commonly used by device manufacturers in the early stages of device production as this tool helps understand the ray paths, predicts theoretical peak intensity (according to LED datasheet optical power output) and provides inputs on ways to optimize the use of reflectors and/or optical components within the device. The tool employs a low cost and quick technique of ray tracing. The software models the propagation of light from the source designed, through the system, and on to the final point of interest. The resulting distribution of rays within the system is used to predict a wide range of light parameters as per the operator’s interest ^33^.

### Governing equation

Ray tracing involves the use of two fundamental properties of a ray – position and direction. The position and direction of the ray in a Cartesian coordinate system is defined in Equation 1 and Equation 2.

$$\vec{r}=\{x,y,z\}\;(1)$$

$$\hat{k}=\{l,m,n\}\;(2)$$

In Equation 1, $\vec{r}$ is the position of the ray and ( *x, y, z*) are coordinates measured in units of length depending on the type of system analysis. In Equation 2, $\hat{k}$ is the direction of the ray and ( *l, m, n*) are the direction cosines of the unit vector that points along the ray. Both these quantities are measured based on local coordinates or global coordinates relative to the reference frame input by the operator. If a ray is propagated by distance *x*, where *x* is the length in SI units, the new coordinates of the ray is given by Equation 3.

$$\vec{r^{\prime }}=\vec{r}+x\hat{k}\;(3)$$

To predict ray path by refraction, reflection or diffraction within the set-up, the tool employs Snell’s law in vector form at the point of intersection with a surface ( Equation 4)

$$n^{\prime }(\hat{N}*\hat{k\prime })=n(\hat{N}*\hat{k})\;(4)$$

Where *N* is the unit normal vector of the surface at the point of contact and *k* is the ray direction cosine vector. The above Equation 4, changes with the kind and type of optical phenomenon detected at the point of intersection between the surface and ray. To optimize and understand the ray path and light system associated with it, the tool has the option for two types of ray tracing -sequential and non-sequential ray tracing. In sequential mode, light rays are limited to propagating from one point to the next and is not flexible for complex systems. Non-sequential ray tracing allows the rays to propagate through the components within the system and allows ray splitting, scattering and reflections to occur during simulation. This method of ray tracing means that there is no specific sequence for movement of rays within the system i.e., the rays may hit any part of the system designed and move in any direction based on the optical phenomena detected at the point of contact by the software ^33^.

### Boundary conditions and material properties

Non-sequential mode of ray tracing has been employed in this study. Contrary to the case of DOM simulations, the media between the LED array and the radiometer need not be included in the simulation to calculate radiation transport. In the case of ZeMax, the body is designed using shapes available on the software, like point sources, ray sources, two angle sources etc. ^33^. In the case of this study, the source has been designed as radial source and the other parts were designed using multiple shape options available on the tool based on the device used to match and replicate the actual device manufactured. Each LED footprint is taken into consideration while designing the device on the tool. Dimensions like the thickness of the LED package, size of the actual light emitting surface, and optical power output from the source is drawn from the datasheet, while other parts are designed according to dimensions provided by ProPhotonix. A screengrab of the modeling stage on the optic studio can be seen in Additional Data (Figure S1).

In the ZeMax user interface, the entire emission radiation pattern can be input in the source properties, ensuring the simulated source irradiation is similar to the manufactured light source. From the datasheet of the LEDs, the radiation pattern has been extracted and used as an input in the tool. The 265 nm and 310 nm LEDs have a viewing angle of 120° and 130°, while 275 nm LEDs have a higher viewing angle of 150°. The ray-tracing simulations, in this study, assumed that LEDs emit light as Lambertian point sources. While this approach is similar and computationally efficient, however it does not fully capture the behavior of LEDs in near-field conditions, wherein the distance between the source and the object is small. Future work could incorporate models that treat LEDs as extended sources to improve accuracy in such scenarios ^41– 43^.

A key parameter that controls the simulated values is optical power. Simulations were conducted, initially, using the power output of the LED (in mW) specified in the LED manufacturers datasheet to understand the theoretical intensities delivered to points of interest. Using radiometric experiments conducted in air, the actual power output of the LEDs has been recalculated to ensure that the model behaves close to actual measurements. This provides an understanding of how the LED behaves within the device controlling the light output. Once this is done, the model can then be used to simulate the presence of a quartz tube and water in front of the source.

A quartz tube is not readily available on the interface and multiple object types were considered before arriving at the use of a cylindrical object type. The quartz tube was modeled using a Boolean technique to simulate the hollow tube and the inner material of the pipe was changed to “ *water*” for water medium simulations while the outer cylinder material was changed to “ *quartz*”. The properties of the quartz tube are as per the quartz library-loaded material properties on the tool. The properties of the tube are as per data derived by Bass *et al*. ^44^. On the tool, the main parameter dictating simulation accuracy is the number of rays within the simulation. For a rough understanding of the light emission and path within the system, a low ray count will work, but for accurate simulations, higher ray count is recommended.

**Figure 4.**
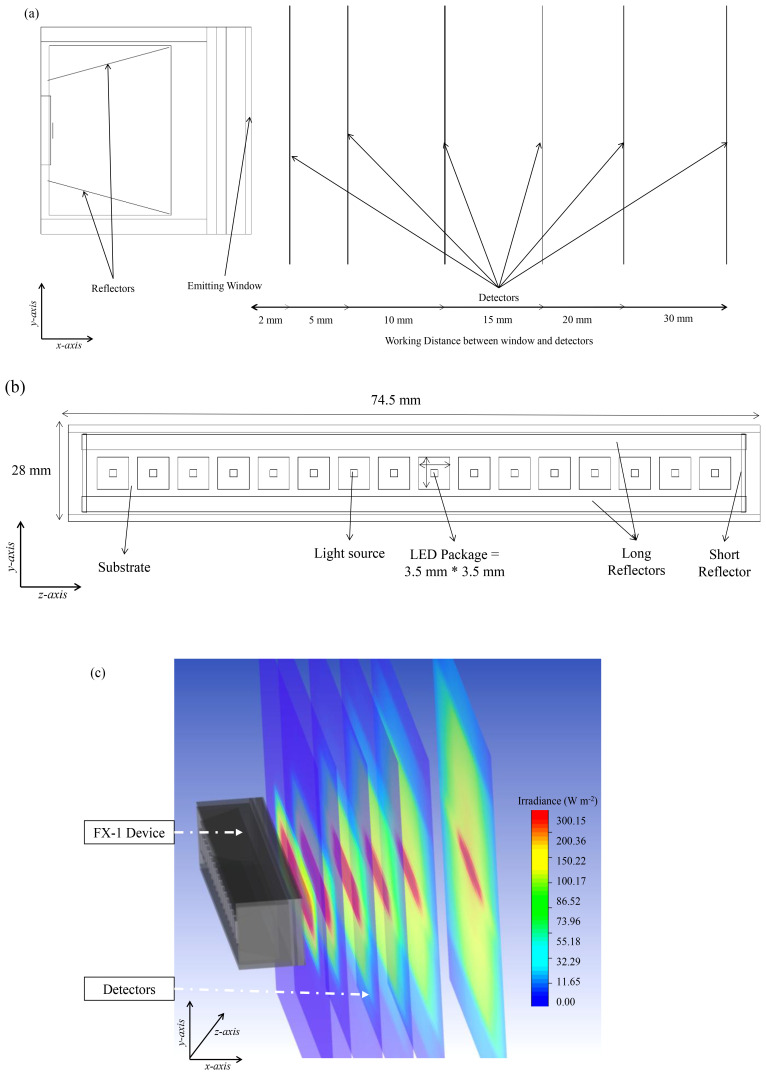
( **a**) Side view of the designed model on ZeMax interface, ( **b**) Top view of the designed model ( **c**) Section view of simulated FX-1 265 Optical Model.

In the case of this study, all simulations have been conducted at 10 ^6^ rays per model simulated. For each simulation, the computation time was approximately 1 hour per wavelength using a workstation with an Intel i7 processor or equivalent and a dedicated GPU. The computing time was primarily dependent on the number of rays and the complexity of the system design. The value of 10⁶ rays per model has been chosen to ensure a high degree of accuracy in modeling the irradiance distribution. While this number was not exactly optimized for computational efficiency, it was chosen based on standard practices in optical simulation. Future work will be needed to investigate the minimum number of rays needed to achieve similar results with reduced computational load. To quantity and analyze the data from light simulations, detector rectangles (here onwards called analytical detectors) are designed within the model. Analytical detectors are created at multiple working distances to simulate and quantify the effect of working distance on the intensity of light from the source. Figure 4a, b shows a sectional view of the 265 nm source designed on the software depicting the light source, the mechanics within the device and detector objects as black lines in the *x* direction. For simulations involving the generation of water medium, the refractive index was changed with each wavelength simulated. The input data for these models have been listed in Table 2.

**Table 2.** Wavelength specific absorption coefficient, refractive index and quantum yield in water ^46, 47^.

Wavelength	Absorption Coefficient (m ^-1^)	Refractive Index	Quantum Yield (ɸ)
265 nm	0.1m ^-1 48^	1.3572 ^46^	1.2281 ^47^
275 nm	0.1m ^-1 48^	1.3540 ^46^	1.2629 ^47^
310 nm	0.01 ^48^	1.3478 ^46^	1.2281 ^47^

The water temperature during the simulations was fixed at 25°C, and the refractive index values were referenced from 45 for this specific temperature. Also, the tool assumes default values based on the material input for values of absorption and scattering coefficient. In this case, the scattering coefficient was set to 10 ^-2^ m ^-1^, whereas the absorption coefficient varied at each wavelength. These coefficients are listed in Table 2. It is worth noting that these values were considered default and not changed throughout the simulations conducted, as the focus was primarily on optical ray paths, and internal reflections rather than volumetric absorption and scattering in the media.

### Characterization using chemical actinometry

Actinometry is a method by which the number of photons in a beam can be measured by use of a chemical system that absorbs the incident radiation in a defined space of a reactor. The method determines the number of photons integrally with time. Reactants used within this chemical system undergo a light-induced reaction for which the quantum yield is known. Quantum yield (Φ(λ)) of a photochemical reaction can be defined as the number of events like molecules formed divided by the number of absorbed photons of that wavelength ^49^. Measurement of the reaction rate allows the calculation of absorbed photon flux ( Equation 5).

$$q_{p}(abs,\lambda )=q_{p}^{o}(1-10^{-A(\lambda )})\;(5)$$

where, $q_{p}^{o}$ is the incident photon flux and A (λ) is the decadic absorbance. There are different chemical systems listed by International Union of Pure and Applied Chemistry (IUPAC) like solid and micro heterogeneous-phase chemical actinometers, gas-phase chemical actinometers and liquid-phase chemical actinometers. Based on the wavelengths studied and the type of set-up, a liquid phase chemical actinometer has been selected. Specifically, potassium ferrioxalate (K _3_[Fe(C _2_O _4_) _3_].3H _2_O) based chemical system has been used for this study. The chemical system is also recognized as the Hatchard-Parker actinometer by IUPAC and is widely accepted as a standard actinometry test for ultraviolet wavelengths. The actinometer has a wavelength range of 250 – 500 nm with a quantum yield (Φ) of 1.25 – 0.9 ^50, 51^. The quantum yield used in calculations for each wavelength has been listed in Table 2.

**Figure 5.**
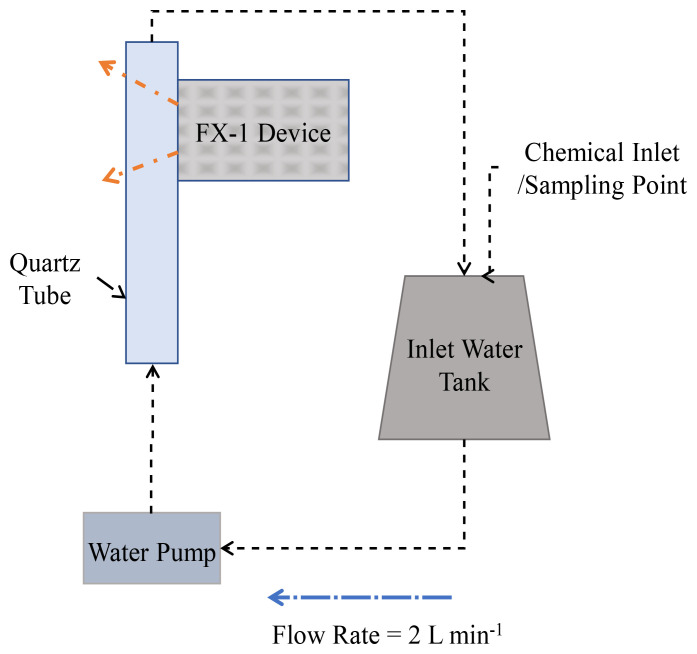
Schematic representation of the set-up used for actinometric experiments.

To characterize the device using chemical actinometry, the UV fixture has been used ( Figure 2a). For each test, only one device of a specific wavelength has been used at a distance of 14 mm away from the center of the quartz tube. A pump with a flow rate of 2 L min ^-1^ has been used to flow the prepared solution through the fixture. Figure 5 is a schematic representation of the set-up used. 2 L of milliQ® water have been used as the base water matrix for these experiments. The experimental procedure is same as used by Hatchard *et al*. ^51^. Tests have been conducted for each wavelength on three separate days, with samples extracted at different time intervals to ensure repeatable and reproducible data is obtained *.*

### Discrete Ordinate Method (DOM) modeling

***Governing Equation.*** Discrete Ordinate Method is one of the available models used to calculate radiation transport implemented in Fluent in the multiphysics ANSYS™ platform ^52^. The method solves the radiative transfer equation over a domain of discrete solid angles. In this technique, the incident radiation is calculated by integrating the radiant intensity along spherical space. The method employs discretization of spatial directions and solves the Radiative Transfer Equation (RTE, Equation (6), ^52^) for each direction. It calculates radiation intensity as a function of absorption, scattering, reflection and emission. The equation describes the conservation of radiative intensity in a direction of space.

$$\frac{dI_{\lambda ,\vec{\Omega }}}{ds}=\underset{Absorption}{\underset{︸}{-k_{\lambda }I_{\lambda ,\vec{\Omega }}}}\underset{OutScattering}{\underset{︸}{-\sigma_{\lambda }I_{\lambda ,\vec{\Omega }}}}\underset{ThermalEmission}{\underset{︸}{+\frac{k_{\lambda }T^{4}}{\pi }}}\underset{InScattering}{\underset{︸}{+\frac{\sigma_{\lambda }}{4\pi }\int_{\Omega^{\prime }=4\pi }p(\vec{\Omega^{\prime }}\to \vec{\Omega })I_{\lambda ,\vec{\Omega }}d\vec{\Omega^{\prime }}I_{\lambda ,\vec{\Omega }}}}\;(6)$$

where $I_{\lambda ,\vec{\Omega }}$ is the intensity of photons with wavelength λ, propagated along direction $\vec{\Omega }$ ; k _λ_ is the volumetric absorption coefficient; σ _k_ the volumetric scattering coefficient; σ is the Stefan-Boltzmann constant; T is the temperature in degree kelvin and $p(\vec{\Omega^{\prime }}\to \vec{\Omega })$ is the phase function that describes the directional distribution of scattered radiation.

The device was designed on Solid Edge CAD software and converted to ANSYS™ Workbench for radiation simulations on ANSYS™ Fluent. For this study, the light has been assumed to be monochromatic. Each LED within the device has been simulated as a flat surface emitting light. The following assumptions have been made before conducting simulations: i) Thermal emission is neglected by setting the temperature to 0 Kelvin, ii) Density and viscosity are considered constant, as per standard material properties, for the wavelength range studied, iii) Absorption coefficient of 0.00074 m ^-1^ and scattering coefficient of 0.00049 m ^-1^ have been assumed for air at the wavelength range (250 nm – 320 nm) from previous studies ^53^.

### Boundary conditions and material properties

**Figure 6.**
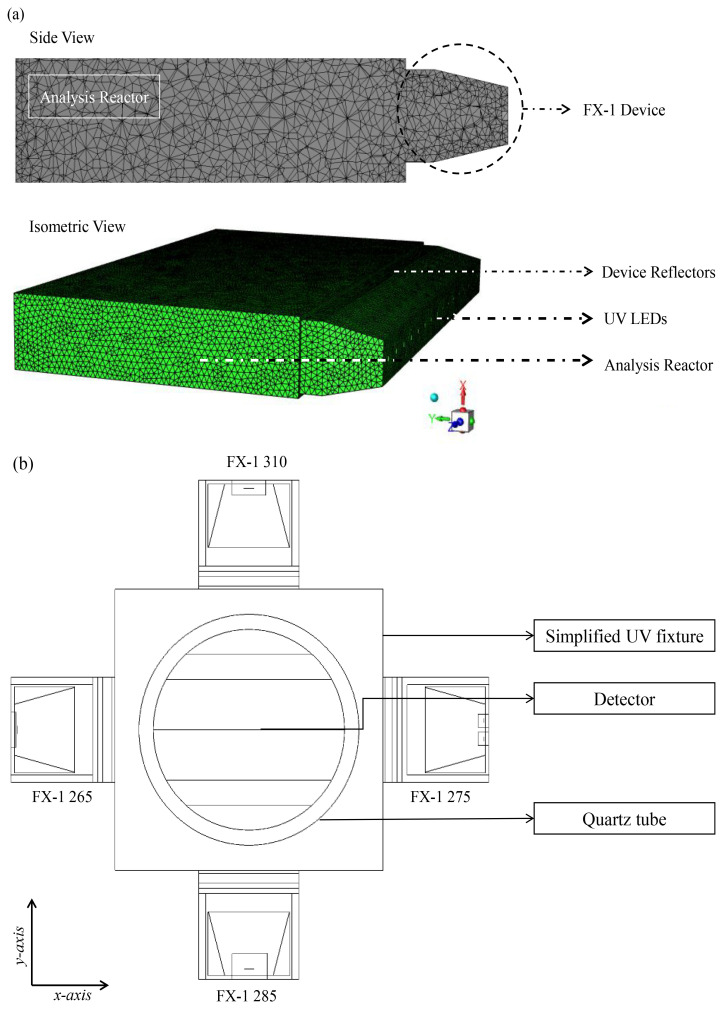
( **a**) Meshed DOM model of FX-1 265, modeled in ANSYS Fluent, ( **b**) Top view of the 4-wavelength complex system.

To simplify simulation time and the number of equations within the model, only the emitting window section (light head) of the device has been modeled (see Figure 6a), with the same dimensions as the experimental system: 76.8 mm × 28 mm with 4 reflectors inside the emitting window. For each wavelength, the LED size and number of LEDs have been modeled as per the supplier design and datasheet specifications. For the study, the reactor has been considered as a simple black box that absorbs any radiation irradiated by the source. The reactor has slightly larger dimensions than that of the emitting window. Before extracting data from the simulations, a grid independence test (mesh sensitivity analysis) has been conducted. A grid independence test was conducted to verify that the solution was not influenced by the mesh density. Several mesh densities were tested, and once the results stabilized beyond a specific number of elements, the final mesh was chosen to ensure computational accuracy and efficiency. For further information, refer to additional data (Table S1, Figure S2–S4).

For simulation on Fluent, the following conditions have been used.

a) Boundary Conditions: (i) Light source – Direct irradiation and beam width as per manufacturer specifications; (120° for 265 nm, 150° for 275 nm and 130° for 310 nm FX-1) (ii) Reflectors –Zero internal emissivity and diffuse fraction; (iii) Reactor, emitting window and LED substrate –100% internal emissivity and diffuse fraction.
b) Refractive index of air has been accounted for each wavelength from literature studies (265 nm – 1.00029777, 275 nm – 1.00029570 and 310 nm – 1.00029023 ^46^).
c) For the light source, angular discretization of 15 x 15 solid angles per octant has been used, enough to capture the LED beam angle.
d) Optimum mesh size from the mesh sensitivity resulted in an average of 417,876 cells per wavelength by use of an inflation mesh at the LED surface and surface mesh for the reactor body. The grid independence test confirmed that the results were consistent across different mesh densities, indicating that the mesh used in the final simulations was sufficient for accurate results.
e) Second order upwind solution method for the DOM model with up to 500 iterations for calculation of the solution.
f) Convergence of numerical solution was ensured by monitoring the scaled residuals to a criterion of at least 10 ^-6^ for discrete ordinates and energy variables.

### Modeling of a 4-wavelength germicidal system

Upon validation of the simulated model using the ray tracing method, a complex system consisting of 4 FX-1 devices operating at different wavelengths was modeled in water medium. The purpose of this model was to determine the contributions of each wavelength to the overall dose received to a water matrix. Therefore, in order to isolate each individual wavelength on the UV fixture, an additional UV source (FX-1 285) consisting of 16 LEDs irradiating at peak wavelength of 285 nm (XST-3535-UV, 40mW, Luminus) was added to the model. The model was modified to replicate the UV fixture seen in Figure 2a. The design was simplified for lower simulation time. The same number of rays (10 ^6^) as mentioned earlier, was used in simulations. Figure 6b depicts the model of the complex system.

## Results and discussions

### Ray tracing and radiometry

**Figure 7.**
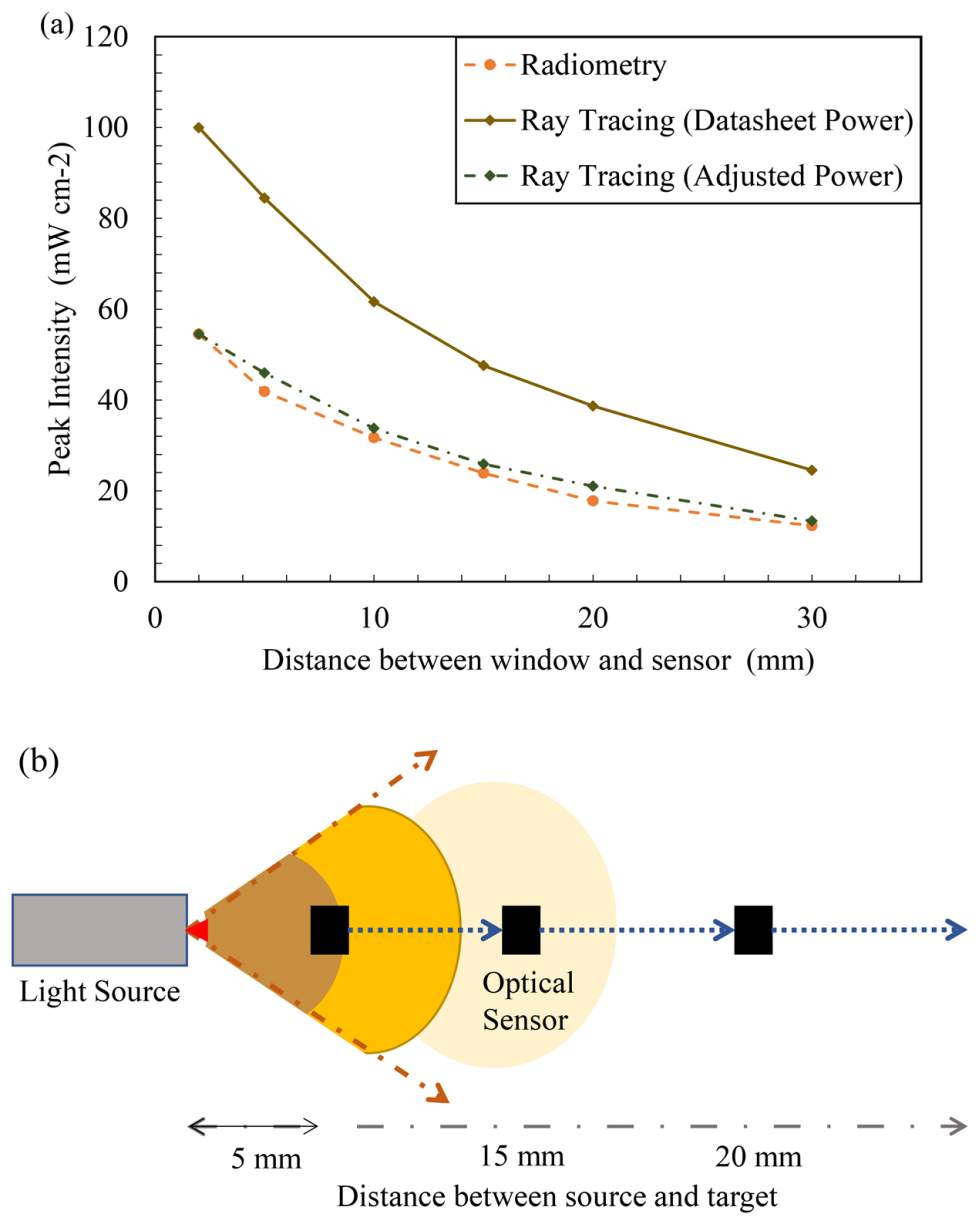
( **a**) Ray tracing data vs experimental data (FX-1 265), ( **b**) Representation of variation of light intensity with distance.

As mentioned earlier, one of the key inputs into the software is the optical power output per LED source designed. To understand how the tool is used in industrial applications, initial simulations have been conducted using the data sheet mentioned optical power output (in mW) of the source. It can be seen in Figure 7a (diamond marker full line), the predicted peak intensity, at multiple working distances. The result predicted by the simulation is higher than the actual peak intensity delivered by the device. The manufacturer mentions that the 265 nm LED and 275 nm LED have an optical power output of 70 mW (at 500 mA) and 8 mW (at 40 mA), respectively, while the 310 nm LED has an optical power output of 50 mW (at 350 mA).

The LED manufacturer conducts tests, on the LED, in a near ideal environment and hence the theoretical peak intensity observed is considerably higher than the actual power output from the device. The actual power output from each LED must be dealt on a case-by-case basis depending on the system and electronics that are driving the light source. LED device manufacturers conduct a rollover test that helps conclude the safe working current and, subsequently, the safe operating power output for higher lifetimes of the device ^54^. The test involves measuring the optical power output from the device with an increase in LED input current until the maximum input current capacity of the LEDs within the device. After a certain input current, any further increase does not result in a significant increase in optical power output. At this stage, any further increase means that the LEDs are generating more heat than light signal, hence are prone to degrade faster. Lighting device manufacturers call this point the rollover point and program the device input current to slightly lower or higher level (depending on safety of other device electronics) for safe operation and long lifetime of the LEDs. In the case of the FX-1’s used, upon rollover tests (refer to additional data for data on rollover tests, Figure S5), the operating current for the devices was set to 317 mA for FX-1 265, 139 mA for FX-1 275 and 437 mA for FX-1 310.

For example, the FX-1 265 rollover test results indicated a LED safe current for the device of 317 mA and corresponding optical power output from each LED of 33 mW. Using this value as the input required for the source, the input power on the software was adjusted to check if the model was behaving the same as the experiments. It can be seen in Figure 7a that the data from the adjusted power simulation (diamond marker dotted line) is very close to the data measured by the spectroradiometer (circle marker broken line) (± 5%). Data on comparison between simulations for other wavelengths can be seen in Additional Data (Figure S6). Comparing the two techniques, it was concluded that the device-based optics designed in the model are very similar to actual device conditions. The base model of the light source can now be used to simulate other conditions and compared with techniques.

For any light source, it is well-established that the total intensity acting on a target area follows Bouguer's law, which describes the relationship between light intensity and distance from the source, as first demonstrated by Pierre Bouguer in his 1729 experiment ^55, 56^. With increase in the distance from the target, the light intensity decreases as the spread of light is wider. The same number of photons emitted by the light emitting surface is spread over a wider area at a longer distance from the source. Figure 7b is a representation depicting how light intensity decreases with increase in distance away from the source. To characterize the light source using a spectroradiometer, working distances of 14 mm to 34 mm away from the source window (in 5 mm steps), as designed within the UV fixture, have been selected for measurements. The selected working distances provide an understanding of the behavior of light in air within the fixture. Radiometric measurements were conducted on 3 separate days to ensure repeatability and accuracy of the obtained data. The experimental error obtained from measurements was low and found to be after the third decimal point. The error was calculated as the standard deviation of all measurements conducted for each wavelength. Although all wavelengths have been measured and tested, only FX-1 265 data has been presented, as the other wavelengths showed similar behavior and were in close agreement with the ray tracing simulation data.

**Figure 8.**
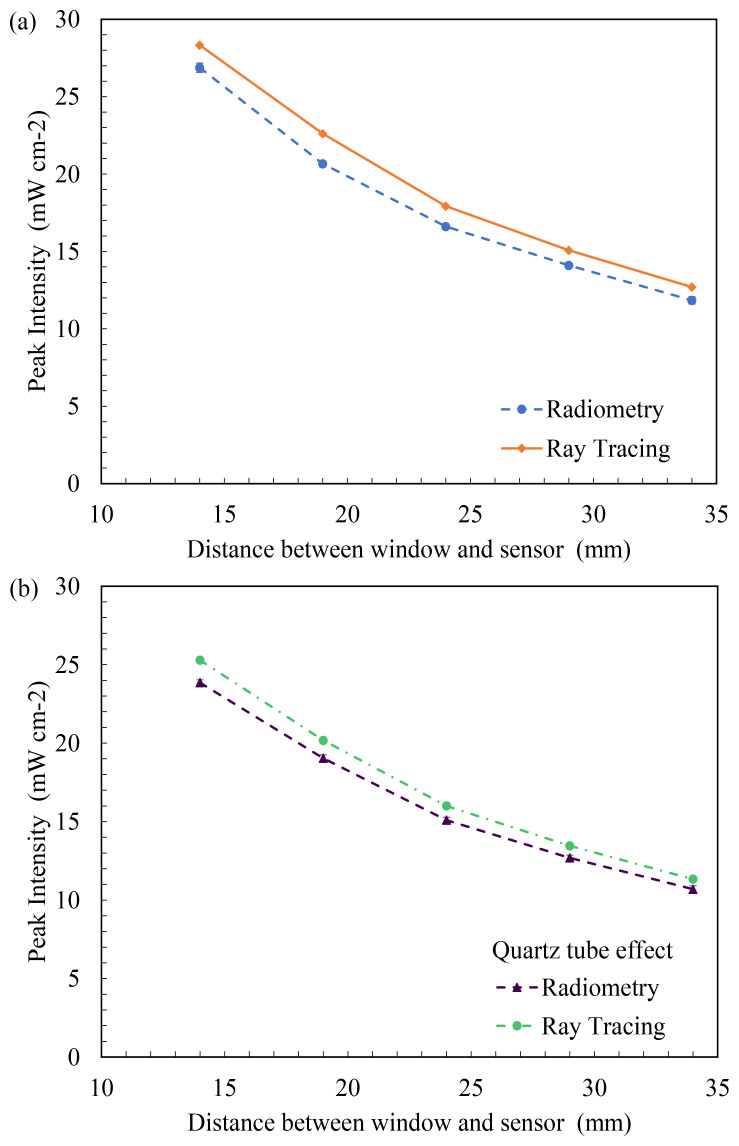
( **a**) Plot of peak intensity vs working distance (in air) and ( **b**) Comparison between radiometry and ray tracing in the presence of a quartz tube in front of the light source for FX-1 265.

Figure 8a plots the peak intensity measured at each working distance versus the working distance between the source emitting window and coupling optic for FX-1 265. It was seen that all the 3 wavelengths selected for this study follow the expected trend of a decrease in light intensity with an increase in working distance for both techniques (Figure S6, S7). Ray tracing simulations can be seen to have the same trend as the radiometric measurements with an average of ±7% difference between the two techniques for 265 nm source, while an average difference of 7% was observed for other two wavelengths. Similar to findings in the literature, the ray tracing technique overestimates the actual light intensity in the simulations. The difference and higher peak intensity observed can be attributed to the fact that in radiometric measurements, light bouncing off the workbench surface is lost while this does not happen in the simulation environment. Although designed as per dimensions and specifications, the design models do not fully replicate the actual environment and hence a difference is expected. Within the X-Y tester, the sensor/coupling optic moves in X-Y direction for a period of time and captures data at each set point and displays light measured at each point at that instant of time which could be another reason to explain the difference between techniques. To summarize the comparison between radiometric measurements conducted in air and the optical ray tracing simulations, Table 3 provides data on measured peak intensities at each working distance for the data seen in Figure 8a. Plots of peak intensity versus working distance for FX-1 275 and FX-1 310 can be found in additional data (Figure S6 and Table S2). Among the three wavelengths under study, relative to the FX-1 265, the FX-1 275 emitted 1.4% lower intensity while FX-1 310 emitted 23% higher intensity at 14 mm working distance away from the center of the system.

**Table 3.** Recorded peak intensity at multiple working distances using radiometry and ray tracing.

Working Distance (mm)	Peak Intensity (FX-1 265) (mW cm ^-2^)					
	Absence of quartz tube		Presence of quartz tube		% deviation in the absence of quartz tube	% deviation in the presence of quartz tube
	Radiometry	Ray Tracing	Radiometry	Ray Tracing		
14	26.87 ± 0.30	28.32	23.85 ± 0.44	25.28	5.39	5.99
19	20.65 ± 0.19	22.60	19.04 ± 0.26	20.18	9.44	5.98
24	16.61 ± 0.16	17.92	15.09 ± 0.22	16.00	7.88	6.03
29	14.09 ± 0.11	15.07	12.69 ± 0.42	13.45	6.95	5.98
34	11.84 ± 0.23	12.69	10.69 ± 0.34	11.33	7.17	5.98

### Effect of Quartz Tube

To analyze the impact of the quartz tube on light irradiated, Figure 8a provides a comparison between the radiometry measurements done in the presence (circle marker broken line) and absence of the quartz tube at different distances from the light source (diamond marker full line) for FX-1 265. The quartz tube reduces the amount of irradiation reaching the target surface by 11%, at a working distance of 14mm away from the center of the quartz tube and follows the same trend as expected. In the same way, the ray tracing simulations resulted in similar data to that of the radiometric measurements, as seen in Figure 8b (triangle marker line). Table 3 summarizes the measured and simulated data from these experiments and can be compared with data measured in the absence of a quartz tube to understand the impact of quartz material on the light irradiated for FX-1 265. In the presence of a quartz tube in front of the source, good agreement can be observed between the two techniques, within ±6% of the radiometric measurements for all the wavelengths studied. Plots of peak intensity versus working distance for FX-1 275 and FX-1 310 can be found in additional data (Figure S7 and Table S3). Compared to FX-1 265, FX-1 275 emitted nearly the same intensity (a difference of about 0.004%), while FX-1 310 emitted 32% higher intensity at 14 mm working distance away from the center of the quartz tube.

As mentioned earlier, this study attempts to analyze and quantify the effect of quartz tube or quartz material on the light emitted. This study employs the use of a custom manufactured quartz tube cut along the length to understand the impact. The tests provided both an understanding of the impact of using a quartz material in front of the light source and an input for the simulations. To better understand the effect of quartz, the manufacturer of the half quartz tube provided the transmission curve of the quartz material used by the tube (see additional data Figure S8). The measurements were conducted, and data was validated to ensure that the loss due to quartz material was well within the range expected as per the material specifications.

**Figure 9.**
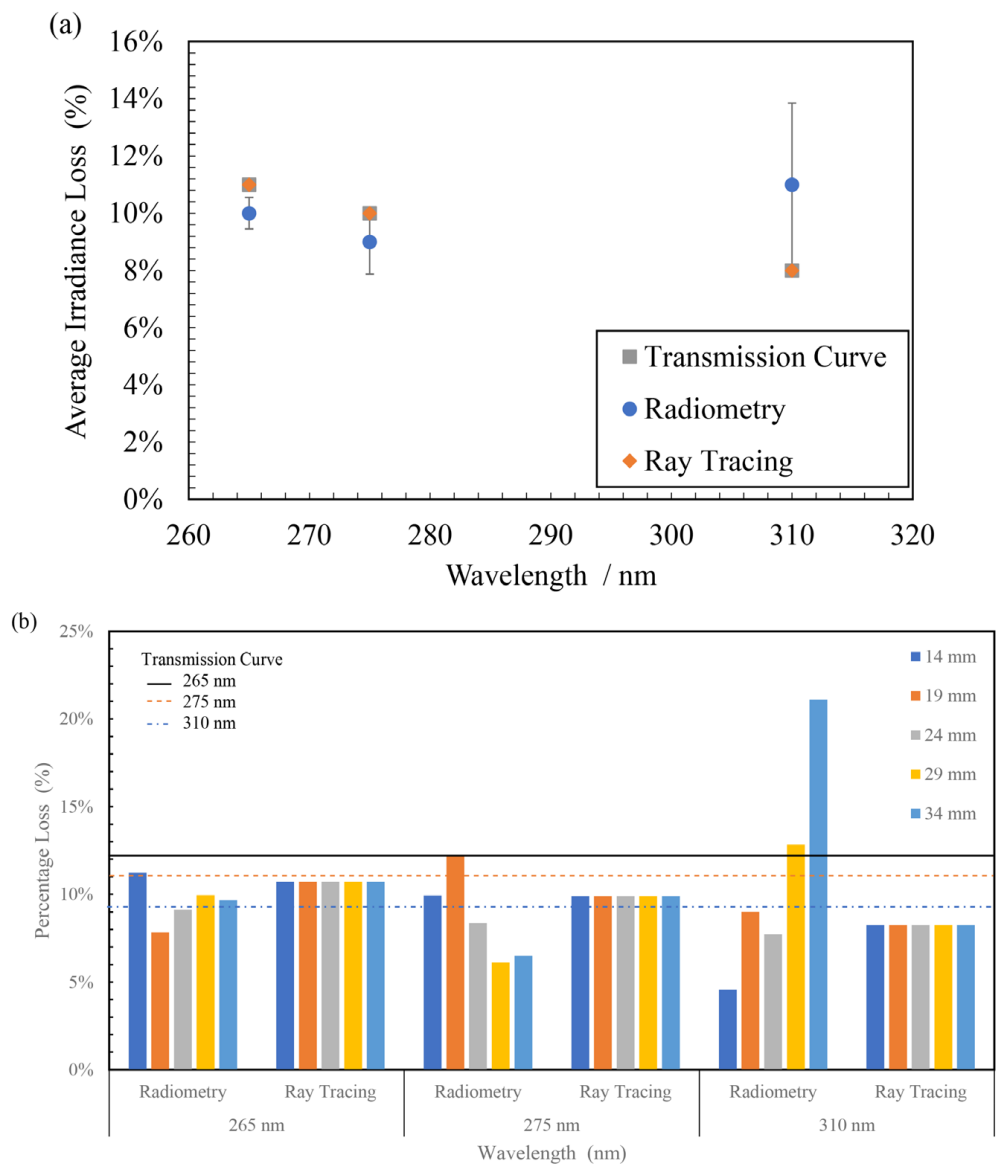
( **a**) Comparison between average light loss at multiple distances in radiometry, ray tracing and transmission curve due to quartz material and ( **b**) Plot of percentage light loss for all wavelengths at multiple working distances due to quartz material.

In Figure 9a, b, the average of all losses observed at multiple working distances in the radiometric measurements have been compared with the ray tracing simulations and the transmission curve obtained from the manufacturer. While the data for FX-1 265 and FX-1 275 can be seen to have good agreement between the techniques and the transmission curve, the error bar and loss for FX-1 310 is high due to the 21% loss of light seen at 34 mm working distance (see Figure 9b). On the ray traces, a constant loss was observed throughout all working distances and all wavelengths. In the optical simulations the light travel within a device or system, the intensity does not vary unless other structures or objects affect the ray trajectory. Therefore, no change in loss is observed with working distances.

The data obtained in the presence and absence of quartz tube ( Table 3) was compared to calculate the amount of light lost as it travels through the walls of the quartz tube. In the case of FX-1 265, an average loss of 10 ± 0.55% was observed with a maximum of 11% loss (at 14mm working distance) and a minimum of 8% (at 19 mm working distance). In the case of FX-1 310, where the highest amount of loss can be observed (21%), this can be attributed to multiple reasons within the measuring system. In Figure 3b, it can be seen that there is a gap between the workbench (that moves the device to multiple working distances) and the measuring sensor (which is on a motor gantry). It is possible at higher working distances that the light is being lost due to the gap and the surrounding environment within the working set-up. Also, in the case of these radiometric measurements on the half quartz tube, data was measured along the line of the tube rather than a rectangular space, which means any light not within the length of the quartz tube, at the instant of time when the sensor captures light signal, has not been measured. Table 4 summarizes the average loss observed with wavelength and data on comparison between the techniques.

**Table 4.** Data on loss of intensity in comparison with the transmission curve.

Wavelength / Technique	Radiometry	Ray Tracing	Transmission Curve
265 nm	10 ± 0.55%	11%	11%
275 nm	9 ± 1.12%	10%	10%
310 nm	11 ± 2.84%	8%	8%

### Discrete Ordinate Method

To further understand the method and tool’s capability, this study conducted DOM to compare and validate the ray tracing-based modeling technique. As discussed earlier, the model has been simulated only in an air-medium for comparison and understanding the advantages and disadvantages of the models.

**Figure 10.**
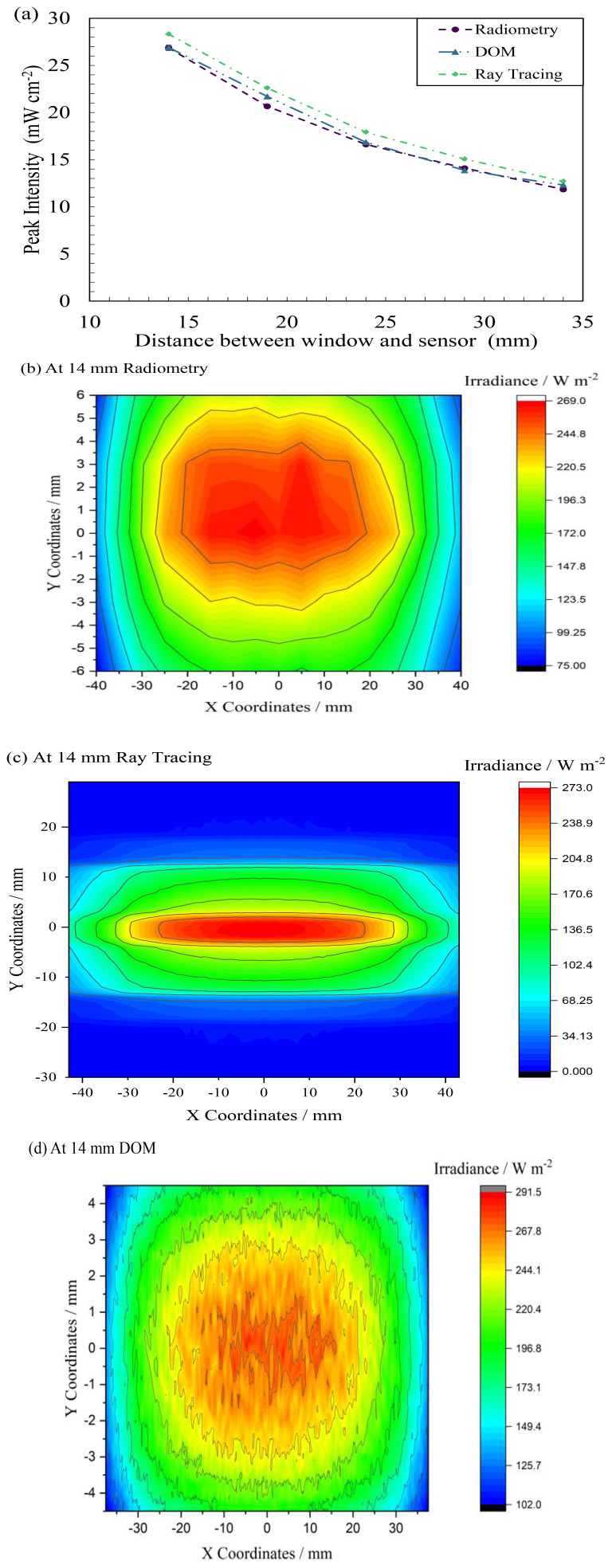
( **a**) Comparison between ray tracing, DOM and radiometry for FX-1 265, Uniformity plot obtained from ( **b**) radiometry, ( **c**) ray tracing interface and ( **d**) DOM.

Table 5 summarizes the data obtained from the simulations for FX-1 265. It has been seen that the data follows the same trend between the two simulations and an average difference of 5 ± 0.86% was seen between the two simulation techniques. This difference could be due to the input value for direct irradiation on the DOM model. The input value, for DOM simulations, has been extrapolated from the radiometric measurements in Figure 8a. While on optical ray tracing, the LED optical power was input for the simulations. Data from DOM simulations can be seen to be closer to the radiometric measurements than that of optical ray tracing simulations (±1.71%). Figure 10a plots the comparison between the two simulation techniques alongside the radiometric measurements. The FX-1 275 and FX-1 310 results can be found in additional data (Figure S9 and Table S4)

**Table 5.** Recorded peak intensity at multiple working distances between simulation tools.

Working Distance (mm)	Recorded Peak Intensity (FX-1 265) (mW cm ^-2^)	
	Ray Tracing	DOM
14	28.32	26.90
19	22.60	21.70
24	17.92	16.84
29	15.07	13.86
34	12.69	12.31

To further understand the difference between the two simulation techniques, the uniformity data has been extracted. Figure 10b – d compares the uniformity plots obtained from the two simulations at 14 mm away from the source along with the uniformity plot from radiometric measurements. While all three techniques display a similar profile, due to the size of the reactor design in DOM, the plot is cut off after the reactor size. In optical ray tracing, the analytical detector size does not impact simulations or simulation time and hence a wider plot could be obtained. The uniformity plot seen in Figure 10d can be seen to be noisy as the data obtained from DOM simulations is a collection of more than 10,000 data points while the radiometry plot is a collection of ~600 high resolution points measured by the radiometer at an instant of time during the measurements. The plot in Figure 10d can be smoothed by extracting data with fewer decimal points from the software. This has not been done so as to provide an overview and comparison between the solutions obtained from respective simulation and experimental techniques.

As seen in Table 5, the ray-tracing simulations tend to overestimate the light intensity at smaller distances. This can be attributed to the assumption of point sources with Lambertian emission patterns, a simplification in ray-tracing methods. However, in near-field conditions, such as in the experiments conducted, LEDs behave more like extended sources, as shown by Moreno *et al*. ^41^ and Valencia-Estrada *et al*. ^42^ and Uppinakudru *et al.* ^43^. Considering LEDs as extended sources could provide better agreement with the radiometric measurements, particularly for short distances between the source and the target, however this was not explored further in this study.

Both the simulation techniques have their respective challenges and disadvantages. Both simulations are time consuming and need an elaborate amount of computational space for each simulation. In DOM, one of the main parameters controlling accuracy and precise simulations is the angular discretization of the light source. An increase in angular discretization increased the simulation time. On ray tracing tool, the main parameter dictating simulations is the number of rays within the simulation. For a rough understanding of the light emission and path within the system, a low ray count will work but for accurate simulations, higher ray count (greater than 10 ^6^) is recommended. Ray-tracing simulations require a balance between accuracy and computational cost. The use of 10⁶ rays in this study provided reliable results, but further investigation into the minimum number of rays needed for equivalent accuracy could optimize the process. Such optimizations would be valuable for reducing computation time, especially in more complex simulations. The simulation time for ray tracing averaged up to 1 hour for high number of rays, whereas on DOM, for high angular discretization (greater than 15 X 15) of the light source, an average simulation time of 2–3 hours was observed.

The input of a number of rays is similar to conducting mesh sensitivity analysis, in DOM. In DOM simulations, it is important that the entire system is built as a solid in a specific space to obtain results. In ray tracing-based simulations, the design of the system is based on an X-Y-Z coordinate system that enables easier design and can be built as per the design and dimensions. In both simulations, the main body can be simplified to reduce simulation time and processing. As in the case of this study, the whole body of the device has not been designed, only the light head section as this is the only part that contributes to simulating light travel. Both methods can simulate the presence of water and air in front of the source, however it was seen that ray tracing method is easier than DOM simulation for both the design and simulation steps. Unlike DOM, each part can be designed independently and hence any issues incurred during or after simulations can be detected specific to the part and not the system as a whole. This helped reduce time for correction and changes whereas on DOM, the time required for understanding the issue and making changes was significantly higher. In simulations where the light source data is of importance, an advantage of ray tracing over DOM simulations is the useful input of the radiation pattern of the light source. In ray tracing, the light source can be programmed as per the manufacturer’s datasheet and optimized for the device design. The ray tracing technique is mainly an optical tool and hence any particle tracking simulations cannot be conducted in this tool while this can be done on DOM ^35^.

To quantify the agreement between the irradiance maps in Figure 10(b) and (d), the correlation coefficient was calculated using eq. 7.

$$r=\frac{\sum (A_{i}-\bar{A})*(B_{i}-\bar{B})}{\sqrt[{2}]{\sum {\left(A_{i}-\bar{A}\right)}^{2}*\sum {\left(B_{i}-\bar{B}\right)}^{2}}}\;(7)$$

Where, *A _i_* and *B _i,_* _represents the pixel values of the two plots, and *Ā* and $\bar{B}$ are the mean values of each plot.

The correlation coefficient was found to be 0.581, indicating a moderate correlation between the two plots. This provides a quantitative assessment of how well the DOM simulations align with the experimental radiometry data, offering insight into the level of agreement between the two methods. It is also worth noting here that there is significant difference in peak irradiance between the DOM results (291.5 W/m ^2^) and the radiometry measurements (269.0 W/m ^2^) in Figure 10(d), at 14 mm distance away. This discrepancy and moderate correlation between the methods may have arisen from a combination of factors, including the level of angular discretization and mesh sensitivity analysis applied in the DOM simulations. DOM results are sensitive to these factors. The sources of these noises have been explored in 57.

In addition to comparing peak intensity, the Root Mean Square Error (RMSE) and Mean Absolute Percentage Error (MAPE) between the experimental radiometry data and the DOM simulation results. The RMSE was found to be 11.01, and the MAPE was 3.51%, indicating a moderate deviation between the experimental and simulated results.

### Characterization using Actinometry

Once the ray tracing model was validated in an air medium, the model was then developed to understand its behavior in a water medium. To do this, multiple changes were made within the designed model discussed earlier.

**Figure 11.**
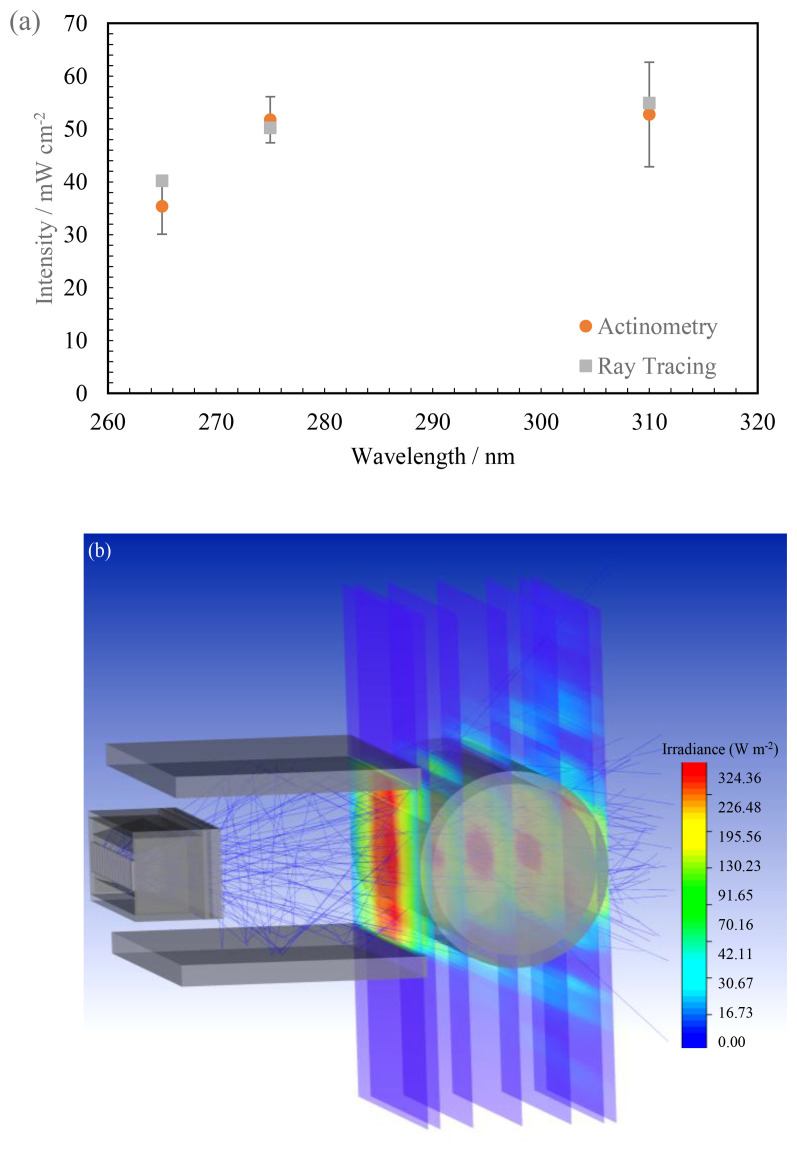
( **a**) Plot of intensity with wavelength between the two techniques, ( **b**) Isometric view of changed ray tracing model.

- To design a tube on the tool, multiple “object types” were considered and tried. A hollow cylinder type object was not readily available and hence to build the quartz tube within the model, a Boolean operation technique was applied. Two cylinders have been designed and using Boolean operations, the inner cylindrical area has been subtracted from the outer solid cylinder to obtain a hollow cylinder of the dimensions of the quartz tube within the UV fixture. In doing so, the software enables the input of two materials for each of the cylinders. The inner cylinder material was changed to “water” while the outer cylinder material was set to “quartz” ^44^.
- While conducting actinometry, the device was placed within the UV fixture and samples were taken. Within the UV fixture, the environment is different to the standard workbench setup seen in Figure 3a. It can be seen that there exist multiple design bodies and/or surfaces, ensuring little light is lost within the system. To ensure that the simulation model replicates the actinometry measurements, two extra design bodies on top and bottom of the light source body (see Figure 11b) have been designed as per the UV fixture model.
- Analytical detectors or measuring points in the model have only been placed within the tube and the software assumes the remaining simulation space as air.
- Refractive index of water within the simulation was changed for each wavelength based on data from literature studies ^46^ and have been listed in Table 2.

Actinometric measurements have been conducted on 3 separate days to ensure reproducible and repeatable data is obtained. The quantum yield used to convert the measured data to mW cm ^-2^ has been listed in Table 2. The technique was subjected to all three wavelengths in this study and only conducted at a working distance of 14 mm away from the center of the quartz tube. This technique was used to validate the ray tracing model and check if the simulation agreed with the measurements. Conducting actinometry for each working distance can be time consuming, whereas the simulation displayed data for all points of interest in less time. Measured data from actinometric measurements can be seen in Table 6 in comparison with the extracted peak intensity data at the center of the quartz tube on the simulated model. It can be seen that both the measured and simulated data are in close agreement with each other i.e., within the error range of the actinometric measurements for all the sources studied (see Figure 11a). The FX-1 275 can be seen to have the closest agreement 51.76 ± 4.37 mW cm ^-2^ in actinometry to 50.22 mW cm ^-2^ on the ray tracing model. The difference between the two techniques is seen to be about 3%. Data on actinometry measurements can be found in additional data (Table S5 and Figure S10).

**Table 6.** Data on comparison between actinometry measurements and ray tracing.

Device	Measured Intensity (mW cm ^-2^)	
	Actinometry	Ray tracing
FX-1 265	34.97 ± 5.51	40.22
FX-1 275	51.76 ± 4.37	50.22
FX-1 310	52.77 ± 9.88	54.93

Upon observing considerable agreement between the two techniques, the effect of water has been quantified using the same model seen in Figure 11b. Simulations have been conducted at multiple working distances within the UV fixture in air and water mediums. Table 7 summarizes the comparison between air medium and water medium for FX-1 265. It can be seen that there is an increase in irradiation at all working distances when light passes through a water medium. It is known that very little light in the ultraviolet range is absorbed by water and hence the increase is predominantly due to multiple optical phenomena occurring within the medium as the light propagates ^23^. As the light passes through the water medium, it undergoes refraction and reflection, meaning multiple rays can be interacting at a given point within the simulation and system. Also due to the design ( Figure 2a) and material (Aluminum) of the UV fixture, very little light is lost and there is a high probability that any light not entering the quartz tube directly is reflected back. Within the set-up, due to supporting structures, light can be seen to reflect off the surface of the fixture and measured within the simulation and in actinometry as seen in the ray traces in Figure 11b.

**Table 7.** Comparison between measured intensity in air and water medium for FX-1 265.

Working distance (mm)	Measured peak intensity. within the set-up (mW cm ^-2^) (Ray tracing)		% Increase in irradiation
	Air Medium	Water medium	
14	25.28	40.22	37.15%
19	20.18	37.42	46.07%
24	16.00	33.91	52.82%
29	13.45	37.76	64.38%
34	11.33	31.32	63.83%

**Figure 12.**
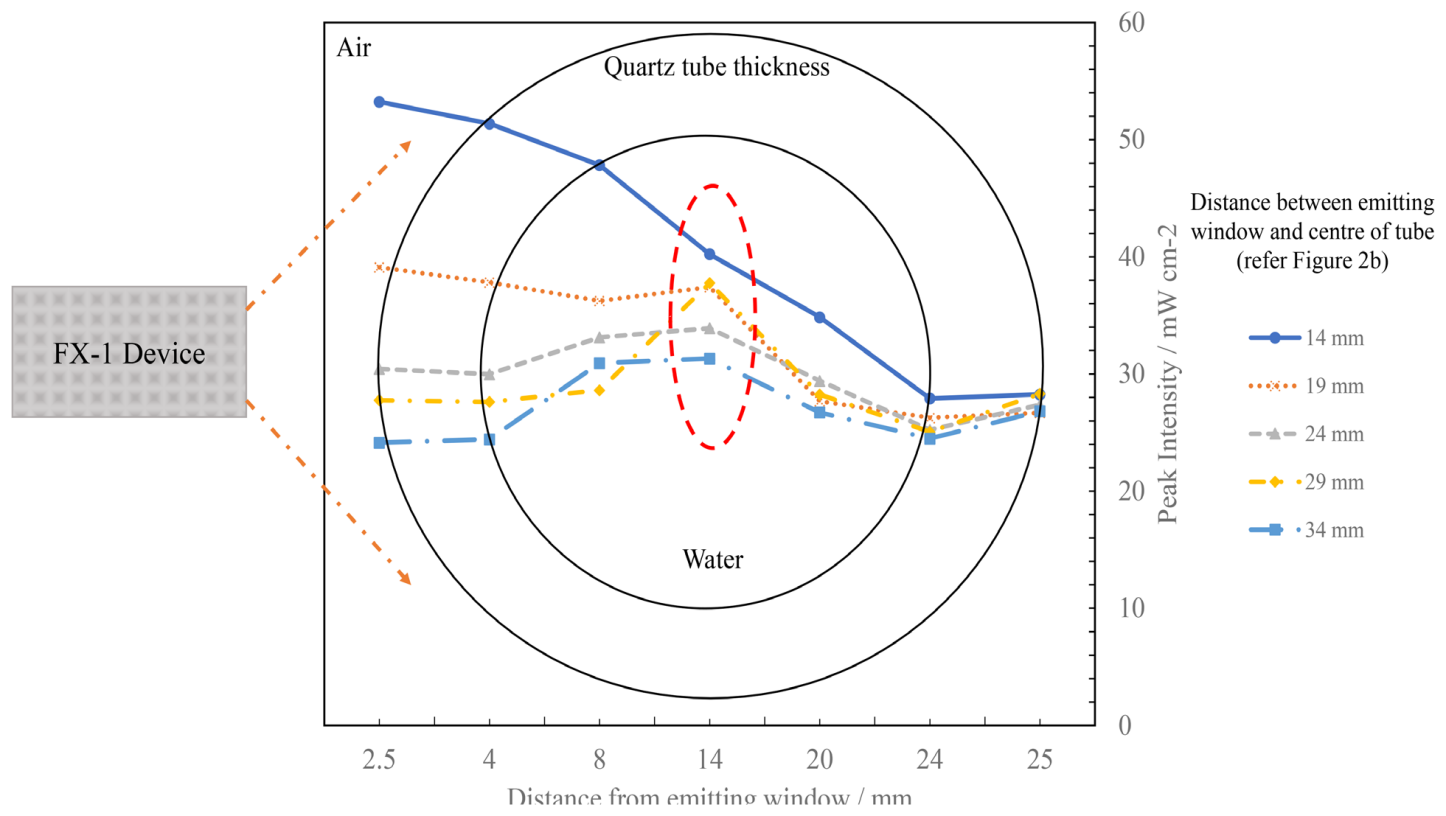
Plot of change in peak intensity as light propagates through the quartz tube within the UV fixture at multiple working distances for FX-1 265.

By creating multiple analytical detectors along the diameter of the quartz tube, the model also enabled an understanding of how the light intensity progresses from the window until the end of the quartz tube (see Figure 11b). Figure 12 traces the measured peak light intensities as the light passed through the system designed at multiple working distances from the emitting window of the device. At 14 mm, the peak intensity profile with working distance follows nearly the same trend as expected (a drop is expected in the maximum peak intensity with distance) but at other working distances the trend observed is significantly different. It can also be seen that once the light enters the quartz tube, the highest intensity simulated is seen in the center of the quartz tube (red circle in Figure 12). This is due to multiple reasons, including reflections from inside the UV fixture body as the distance increases between the source and tube, total internal reflections in water, light reflecting from opposite walls of the fixture and due to the optical phenomenon occurring as the light passes through the thickness of the quartz tube (3 mm).

### Analysis of a 4-wavelength germicidal system

**Figure 13.**
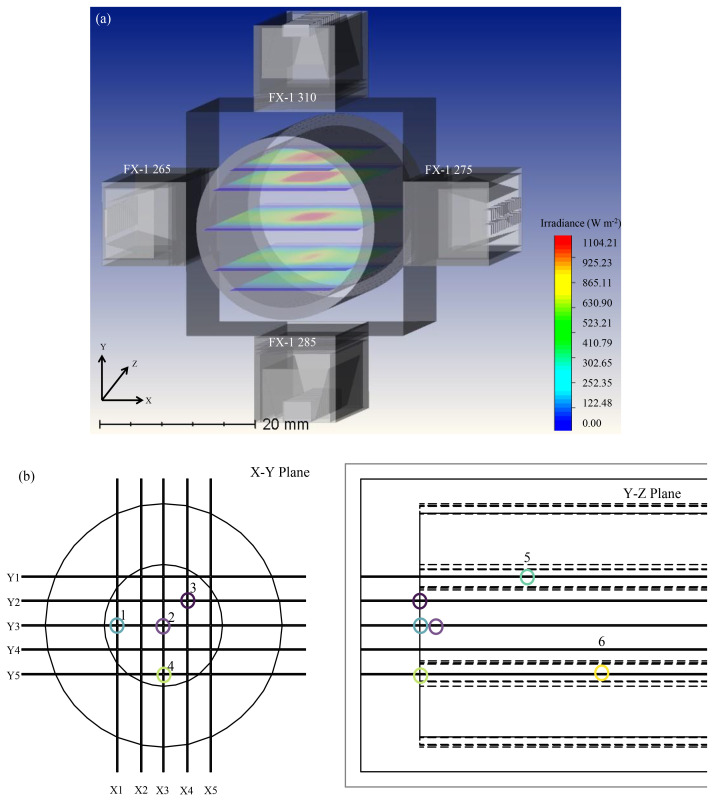
( **a**) Ray tracing model for a complex system involving 4 FX-1’s operating at different wavelengths and ( **b**) Depiction of points of interest in a complex system.

From previous sections, it is clear that the ray tracing tool estimates intensity values within the error ranges of validated and known methods in the literature. In the case of a complex system involving more than 1 wavelength, experimental methods do not provide any information or data on the contribution of individual wavelengths to the overall intensity measured by the respective method. It was observed that ray tracing provided this data. To design a complex system involving multiple wavelengths and devices, the tool allows the use of mirror and rotation functions to reduce modeling time in the case of this study. To simplify the amount of time taken for simulations and to better understand the data observed, two simulations were conducted. The first simulation is as seen in Figure 13a where the analytical detectors are parallel to the *x-axis* to obtain data from FX-1 310 and FX-1 285. For the second simulation, the analytical detectors were moved parallel to *z-axis* to obtain data from FX-1 265 and FX-1 275. The data from the two simulations were combined to provide information on light propagation within the tube. For example, consider points 1–6 in Figure 13b, Table 8 summarises the data observed in these simulations. The simulation time for such a complex model was seen to be about 3–4 hours. This simulation provided insights on amount of light and wavelength reaching points of interest within the tube.

**Table 8.** Simulated intensity in water at different points within the tube.

Point number ( Figure 13b)	Total intensity (mW cm ^-2^)	Spectral intensity in mW cm ^-2^ (% )			
		FX-1 265	FX-1 310	FX-1 275	FX-1 285
1	135.38	50.63 (37%)	20.62 (15%)	13.78 (10%)	50.35 (37%)
2	199.60	40.45 (20%)	54.66 (27%)	50.22 (25%)	54.27 (27%)
3	147.64	37.25 (25%)	35.22 (24%)	29.80 (20%)	45.36 (31%)
4	160.03	46.55 (29%)	17.43 (11%)	34.65 (22%)	61.40 (38%)
5	131.11	16.35 (12%)	44.26 (34%)	27.55 (21%)	42.95 (33%)
6	111.92	22.68 (20%)	10.45 (9%)	27.37 (24%)	51.41 (46%)

Ray tracing has been seen to be effective and efficient in understanding the path of light as it travels through the system. Optimizing a system needs to be dealt on a case-by-case basis and specific to the design of the system. For germicidal systems, it has been known across literature that a range of 250–270 nm is optimal for the effective disinfection of water ^58^. For example, in the case of this study, using data from primary models in air and water, simulated data provided inputs on the behavior of each wavelength within the designed system. When irradiated at the same time, each wavelength contributed a certain percentage irradiation to the overall intensity. In Table 8, it can be seen that at point 2 (center of the quartz tube), FX-1 285 irradiates the majority of the total irradiance reaching the point followed by the FX-1 310 device. To optimize the system, to achieve effective and efficient disinfection of water, FX-1 285 and FX-1 310 can be moved within the UV fixture to 24 mm away from the center of the quartz tube, enabling a reduced contribution of the respective wavelengths to the total irradiation (24.82% and 20.06% respectively). Depending on the type of microorganism being evaluated and disinfected, the germicidal systems’ effectiveness can be enhanced.

It is worth noting that the above study has been conducted for a static model system, however there are other factors such as flow state, water matrix condition that can directly influence the light attenuation process that need to be considered for further studies. At the same time, the effect of quartz material needs to be studied in further detail by evaluating factors such as the effect of the thickness of the tube, material properties and design.

## Conclusion

In summary, the present study reports for the first time the application of an optical ray tracing method for the prediction of irradiation reaching the point of interest as the light propagates through water in a germicidal system. The optical ray tracing modeling method was compared with radiometry, DOM and ferrioxalate actinometry. The results in comparison to these techniques have been seen to be in close agreement (±6%). This proved that the proposed method can be used to overcome major challenges faced during measurement and simulation of irradiation in water. The study also quantifies the effect of quartz material on irradiations in the UV range of light spectrum and observed a decrease of light intensity by 10 ± 0.55% for FX-1 265. The comparison between light transmission curve for fused quartz material used in this study provided an understanding of light behavior at multiple working distances as it passes through the 3 mm thickness of the tube. A constant light loss was observed in simulations, whereas a variable light loss was seen in radiometric measurements. The study also validated measurements in water using ferrioxalate actinometry and provided an understanding of the increase in light intensity in water medium due to total internal reflections and scattering of light in water. The study found an average 52% increase in light intensity across multiple working distances in water for FX-1 265 with respect to air medium in the presence of quartz tube. The method was further used in a 4-wavelength complex system and enabled a better understanding of the system designed. The data obtained showed individual wavelength contributions at multiple points of interest within a complex system. Although the predictions of radiant intensities by optical ray tracing simulations are higher than experimental and other simulation methods, differences are within the error range. Each application needs to be worked on a case-by-case basis. In conclusion, the method provides a valuable understanding of how the light source propagates through the system, how to optimize the light irradiation within the system designed, and the difference between air and water-based systems.

## Data Availability

Repository: https://doi.org/10.5281/zenodo.10054947 This dataset contains all data published in this article. Supplementary information for this article can be found at https://doi.org/10.5281/zenodo.10055089.
