# Supplementary material for: Comparison of radiant intensity in aqueous media using experimental and numerical simulation techniques
Source: Open Res Eur. 2024 Oct 18;4:18. Originally published 2024 Jan 11. [Version 2] doi: 10.12688/openreseurope.16812.2 (PMC11109699; doi:10.12688/openreseurope.16812.2)
Supplement: Peer review history

# Reviewer report: Reviewer response for version 2

**DOI:** 10.21956/openreseurope.20201.r45293

**Published:** 2024-12-05

**Authors:** Sainath A Waghmare, Ramón Pujol Nadal

**Affiliations:** 1 Department of Industrial Engineering and Construction, University of the Balearic Islands(Ringgold ID: 16745), Palma, Balearic Islands, Spain; 2 MGM's College of Engineering and Technology, Navi Mumbai, Maharashtra, India; 3 IIT Bombay, Mumbai, Maharastra, India

The authors have implemented all the corrections and suggestions. The manuscript is more readable and becomes helpful literature in this research area.

Is the study design appropriate and does the work have academic merit?

Yes

Is the work clearly and accurately presented and does it cite the current literature?

Partly

If applicable, is the statistical analysis and its interpretation appropriate?

Partly

Are all the source data underlying the results available to ensure full reproducibility?

Partly

Are the conclusions drawn adequately supported by the results?

Yes

Are sufficient details of methods and analysis provided to allow replication by others?

Yes

Reviewer Expertise:

Ray Tracing, Optical Analysis, Design, CAD

We confirm that we have read this submission and believe that we have an appropriate level of expertise to confirm that it is of an acceptable scientific standard.

# Reviewer report: Reviewer response for version 1

**DOI:** 10.21956/openreseurope.18165.r39511

**Published:** 2024-05-11

**Authors:** Sainath A Waghmare, Ramón Pujol Nadal

**Affiliations:** 1 MGM's College of Engineering and Technology, Navi Mumbai, Maharashtra, India; 2 IIT Bombay, Mumbai, Maharastra, India; 3 Department of Industrial Engineering and Construction, University of the Balearic Islands(Ringgold ID: 16745), Palma, Balearic Islands, Spain

The authors have conducted an in-depth optical analysis of the germicidal system to determine the light intensity at the point of interest. The ray tracing method is used to study the light behavior in aqueous media, followed by the Discrete Ordinate (DO) method to support the conclusion. Further, an experimental analysis validated the results using radiometry and actinometry techniques. The manuscript is well-written and organized. The results are promising and would be helpful literature in this domain. However, the manuscript needs a minor revision to improve quality and clarity for readers.

1. Background: The first line does not describe the actual problem faced by academia and industry. The authors must clearly specify the study's aim and the problem's definition in the abstract.
2. Figure 4 (a) and (b): The quality and text font can be improved to make it more readable.
3. In ray tracing analysis, the authors used 10 ^6^ rays. Did the authors optimize the minimum number of rays used for the simulation?
4. Figure 6 (a) and (b): What type of mesh used for Ansys simulation? A structured mesh would have stood better for the geometry given. Has the grid independence test been performed for mesh?
5. Authors must specify the percentage deviation in both theoretical and experimental results and sources of result deviations.
6. The comparison of different methods in terms of optical calculations (Figure 10), which relies on comparing peak intensity. Another statistical indicator would be more appropriate in this representation.
7. The work is solely independent. The authors compare their numerical and experimental work with each other. A typical comparison with previous literature and outcomes would strengthen the conclusion.

Is the study design appropriate and does the work have academic merit?

Yes

Is the work clearly and accurately presented and does it cite the current literature?

Partly

If applicable, is the statistical analysis and its interpretation appropriate?

Partly

Are all the source data underlying the results available to ensure full reproducibility?

Partly

Are the conclusions drawn adequately supported by the results?

Yes

Are sufficient details of methods and analysis provided to allow replication by others?

Yes

Reviewer Expertise:

Ray Tracing, Optical Analysis, Design, CAD

We confirm that we have read this submission and believe that we have an appropriate level of expertise to confirm that it is of an acceptable scientific standard, however we have significant reservations, as outlined above.

# Reviewer report: Reviewer response for version 1

**DOI:** 10.21956/openreseurope.18165.r39512

**Published:** 2024-05-10

**Authors:** Jorge Garcia-Marquez, Camilo Valencia-Estrada

**Affiliations:** 1 Laboratoire National de Métrologie et d’Essais,, LNE, Pôle Photonique-Energétique, Trappes, France; 2 Photonics, Oledcomm, Vélizy, Île-de-France, France

This paper compares numerical and experimental data on radiant intensity in aqueous media. 

 The authors' rigorous approach involved a conscious assumption based on the LED manufacturer datasheet (page 9), followed by experimental measurement of the power output of LEDs. This data was then used as input optical power for a set of simulations. It is important to note that LEDs do not necessarily have the radiating pattern shown in datasheets; they are primarily designed for lighting purposes and not as an absolute reference in scientific studies. 

 On section **Results and discussions** (page 13 last paragraph),the authors say that: “...it is understood that the total intensity acting on a target area is a function of the light-emitting source and the distance between the source and the target [53].”

 The function of distance for a radiating light source is known as Bouguer's law: [1] J.-E. Morère, La photométrie : les sources de l'Essai d'Optique sur la gradation de la lumière de Pierre Bouguer 1729, dans Revue d'histoire des sciences et de leurs applications 18, n°4, 337-384 (1965). 

 Please remove the term "...variance of light intensity with distance" from Fig. 7 (b). Variance evokes a dispersion measure. The proposed term is "Variation of light intensity with distance." 

 It was Pierre Bouguer's experiment.

 How much computing time is needed to simulate 10 ^6^ rays (page 10)?

 Ray-tracing methods assume LED emissions are Lambertian and LEDs are point sources. For experiments where the distance between the source and the object is small near field), as in this experiment, the radiometric results obtained in Figure 8 match better when LEDs are considered extended sources. (See, for instance: I. Moreno, “LED irradiance pattern at short distances,” Appl. Opt., vol. 59, no. 1, pp. 190–195, Jan. 2020 Ref [1], and J. C. Valencia-Estrada, B. Béchadergue, and J. García-Márquez, “Full-field radiant flux distribution of multiple tilted flat Lambertian light sources,” *IEEE Open Journal of the Communications Society*, vol. 1, pp. 927-942, 2020 Ref [2].).

 The results in Table 5 (page 17) confirm our statement. 

 We are interested in viewing the correlation coefficient between maps (b) and (d) in Fig. 10. The authors may use the equation (65) in the article referenced as [3] here above. 

 Table 7 shows that Fresnel loss reduces as the refractive index difference between quartz and water is smaller than that of quartz and air. However, how did the authors measure the water refractive index? The water refractive index decreases with increasing temperature. 

 ​​The experiment and the simulations were meticulously prepared, ensuring the reliability of the results. The article is clear.

Is the study design appropriate and does the work have academic merit?

Yes

Is the work clearly and accurately presented and does it cite the current literature?

Yes

If applicable, is the statistical analysis and its interpretation appropriate?

Not applicable

Are all the source data underlying the results available to ensure full reproducibility?

Yes

Are the conclusions drawn adequately supported by the results?

Yes

Are sufficient details of methods and analysis provided to allow replication by others?

Yes

Reviewer Expertise:

Instrumentation, Metrology, Radiometry, Photometry, Geometrical Optics, Difraction, Interference, and Polarisation.

We confirm that we have read this submission and believe that we have an appropriate level of expertise to confirm that it is of an acceptable scientific standard.

## References

[1] : LED irradiance pattern at short distances. *Appl Opt* .2020;59(1) : 10.1364/AO.59.000190 190-195 10.1364/AO.59.000190 32225287

[2] : Full Field Radiant Flux Distribution of Multiple Tilted Flat Lambertian Light Sources. *IEEE Open Journal of the Communications Society* .2020;1: 10.1109/OJCOMS.2020.3008989 927-942 10.1109/OJCOMS.2020.3008989

# Reviewer report: Reviewer response for version 1

**DOI:** 10.21956/openreseurope.18165.r38767

**Published:** 2024-04-05

**Authors:** Wenjun Ge

**Affiliations:** 1 Oak Ridge National Lab, Oak Ridge, USA

The research holds promise in bridging experimental measurements with numerical simulations in aqueous media. The paper is original and well-written.

 However, the study's main objective appears unclear. Can the authors restate the main goal of this study? It shouldn't necessarily involve comparing different RTE solvers (DOM vs Monte Carlo), a task proving challenging in this research.

 While the experimental methodology and results are clear, the simulation outcomes fall short. For this specific problem, DOM and Monte Carlo should yield similar results, and comparing the experiment with either the DOM or Monte Carlo outcome should suffice. However, neither the DOM nor Monte Carlo results meet expectations, which makes the comparison less meaningful.  

 Here are my major concerns:

1. What are the absorption and scattering coefficients exactly used for the ray tracing simulation? The authors primarily focus on software utilization rather than its actual functionality.
2. The results are not consistent shown in Figure 10. Peak intensities in Fig. 10(a) closely match for the experiment, DOM, and MC results, but peak irradiance from DOM in (d) (291.5 W m-2) significantly exceeds radiometry measurement (269.0 Wm-2) shown in (b) at 14 mm. Also, the regular DOM solution doesn't typically exhibit such noises, except for ray effects, which should be distinguishable. Moreover, Monte Carlo results should be more accurate compared to DOM with certain noise levels under the given number of rays tracked. The standard deviation plot of the MC is needed.
3. Does the author intend to improve the modeling or algorithm of the commercial software or to capture the main physics? To enhance the study, the authors should narrow its scope, focusing on comparing one method of radiation simulation with experimental data while including additional simulations in supplementary material if necessary. The simulation can provide the physics insights where the measurement is difficult, and the experiment measurement can guide/correct the simulation parameters. From my view, the authors already have very good experiment data and physics insights from the simulation. To improve the credibility of simulations, it is better to provide some level of verification for either the DOM or MC. Verifying results with simpler problems, similar to the 1-D homogeneous case described in Ge et al. [1], can verify the RTE simulation capabilities effectively.

Is the study design appropriate and does the work have academic merit?

Partly

Is the work clearly and accurately presented and does it cite the current literature?

Partly

If applicable, is the statistical analysis and its interpretation appropriate?

Partly

Are all the source data underlying the results available to ensure full reproducibility?

Partly

Are the conclusions drawn adequately supported by the results?

Partly

Are sufficient details of methods and analysis provided to allow replication by others?

Partly

Reviewer Expertise:

Radiative transfer

I confirm that I have read this submission and believe that I have an appropriate level of expertise to confirm that it is of an acceptable scientific standard, however I have significant reservations, as outlined above.

## References

[1] : Comparison of spherical harmonics method and discrete ordinates method for radiative transfer in a turbulent jet flame. *Journal of Quantitative Spectroscopy and Radiative Transfer* .2023;296: 10.1016/j.jqsrt.2022.108459 10.1016/j.jqsrt.2022.108459
